# Chemical Reaction Network Theory elucidates sources of multistability in interferon signaling

**DOI:** 10.1371/journal.pcbi.1005454

**Published:** 2017-04-03

**Authors:** Irene Otero-Muras, Pencho Yordanov, Joerg Stelling

**Affiliations:** Department of Biosystems Science and Engineering and Swiss Institute of Bioinformatics, ETH Zurich, Zurich, Switzerland; University of Illinois at Urbana-Champaign, UNITED STATES

## Abstract

Bistability has important implications in signaling pathways, since it indicates a potential cell decision between alternative outcomes. We present two approaches developed in the framework of the Chemical Reaction Network Theory for easy and efficient search of multiple steady state behavior in signaling networks (both with and without mass conservation), and apply them to search for sources of bistability at different levels of the interferon signaling pathway. Different type I interferon subtypes and/or doses are known to elicit differential bioactivities (ranging from antiviral, antiproliferative to immunomodulatory activities). How different signaling outcomes can be generated through the same receptor and activating the same JAK/STAT pathway is still an open question. Here, we detect bistability at the level of early STAT signaling, showing how two different cell outcomes are achieved under or above a threshold in ligand dose or ligand-receptor affinity. This finding could contribute to explain the differential signaling (antiviral vs apoptotic) depending on interferon dose and subtype (*α* vs *β*) observed in type I interferons.

## Author summary

Type I interferons (IFNs) regulate a variety of cell functions, exhibiting, amongst others, antiviral, antiproliferative and immunomodulatory activities. Due to their anticancer effects, type I IFNs have a long record of applications in clinical oncology. It is still an open question how type I IFNs generate so diverse signaling outcomes by activating the same receptor at the cell membrane and triggering the same JAK/STAT pathway. It has been experimentally shown that differences in ligand affinity towards the receptor, IFN dose and receptor density are translated into different activities, but the underlying mechanisms of differential responses remain elusive. Looking for potential cell decision processes that could help answering this question, we explore the capacity for bistability at different levels of the IFN pathway. The search for bistability sources in interferon signaling is performed within the framework of Chemical Reaction Network Theory, by adapting previous results to the specific context of signaling pathways. Surprisingly, we find a source of bistability already at the early STAT signaling level. As a result, we show that the pathway has the capacity to translate a difference in affinity or IFN dose into a binary decision between High/Low or Low/High activation profiles of two IFN transcription factors (ISGF3 and STAT1-STAT1 homodimers) responsible for the upregulation of two different families of interferon stimulated genes: ISRE and GAS.

## Introduction

### Multistability in signaling

Molecular switches play an important role in cell signaling. It is known that transcriptional switches control differentiation decisions [1] and major developmental signaling pathways use different mechanisms to switch from transcriptional repression to activation of target genes [2]. A system operating as a *switch* responds in an *all-or-none* fashion to a graded stimulus. Instead of a mere ultrasensitive response (sigmoid input-output relationship steeper than the Michaelis–Menten type [3]), *switch-like* systems undergo a transition between two discrete outcomes, often accompanied by hysteresis. In the context of mathematical models of ordinary differential equations (ODEs), switch-like behavior is captured by the nonlinear phenomenon of bistability where two stable steady states coexist for a certain range of the model parameters. A bistable system can switch between two different stable steady states in a threshold dependent manner, producing a sharp change in the output as a response to a gradual change in a stimulus or control parameter. Reaction networks may also have more than two different steady states, as it is the case for multi-site phosphorylation systems [4].

Paradigmatic examples of bistable switches in signaling include the cyclin dependent kinase network that controls the cell cycle [5–8], the transcriptional switch responsible for stem cell fate decision (self-renewal or differentiation) [9], the pheromone sensing MAPK pathway in *S*. *cerevisiae* [10] and the lysis/lysogeny switch in the *λ*-phage [11]. Recent studies also suggest important roles of bistability in several malfunctions and diseases. To name a number of examples, Kafsack et al. [1] discovered a transcriptional switch controlling a differentiation decision in protozoan malaria parasites; Rieger et al. [12] explain through bistability the threshold phenomena in protein aggregation (neurodegenerative disease can originate from the misfolding and aggregation of proteins); Alam and Gorska [13] speculate that ERK1/2 bistability serves as a signaling memory for epithelial priming of the immune system in chronic asthma, and Shiraishi et al. [14] propose a large-scale analysis of network bistability for various human cancers to identify genes that can potentially serve as drug targets or diagnostic biomarkers.

### Multistability detection and Chemical Reaction Network Theory

In the process of developing models of cell signaling pathways the question frequently arises whether a signal transduction mechanism (either the complete pathway or a submodule) is capable of multiple steady states. Bifurcation diagrams are useful to study the qualitative and quantitative behavior of equilibria when appropriate parameter and steady state values to start the analysis are provided. However, standard continuation/bifurcation tools are not suitable in practice if, as in the case of real modeling problems in cell signaling, the systems are high dimensional and there is inadequate information *a priori* about parameter values.

Chemical Reaction Network Theory (CRNT) has been postulated as a promising framework for the qualitative understanding of complex biological systems from structural properties [15–17] and, in particular, for linking structural properties of reaction networks with the capacity for multiple steady states [18–20]. The two main bodies of classical CRNT theory (so called deficiency-oriented and injectivity oriented, see [21, 22] and [18, 23, 24] for examples of each), as well as recently developed methods [25–28], rely on properties of the underlying network structure and provide useful results without any specification of the kinetic constants or steady state values. A theory based on analysis of subnetworks and atoms of multistationarity has been developed in [29, 30]. Algebraic approaches such as Gröbner basis have been also successfully applied to chemical reaction systems [31–34]. In case we have experimental evidence of bistability (for example, in the form of hysteretic dose response curves) we can exploit CRNT results to get insight into the network’s potential qualitative behavior and obtain quantitative information regarding the parameters [35]. However, CRNT results are usually illustrated by theoretic examples *ad hoc* and the application to the modeling process of biological pathways is still scarce (a recent work using CRNT in Wnt signaling by MacLean et al. [36] is a notable exception).

We aim to provide methods that exploit the structure of chemical reaction networks to allow, in combination with bifurcation analysis, for easy and effective detection of multistationary behavior in signaling pathways. To this purpose we develop, within the framework of chemical reaction networks, sufficient conditions for the existence of a saddle-node, taking into account appropriate assumptions in cell signaling settings. Optimization is used to search efficiently through the state-parameter space providing (in case a saddle-node is found) the state and parameter values from which to start a bifurcation diagram using standard continuation/bifurcation tools. If the system is multistationary (i.e. the saddle-node is a saddle-node bifurcation), two equilibrium branches are automatically computed. In presence of mass conservation we exploit previous (deficiency-oriented) work by Otero-Muras et al. [37]. For open systems without mass conservation our approach is based on (injectivity-oriented) results originally developed in the context of continuous flow stirred tank reactors by Craciun and Feinberg [23]. Both approaches cope with common characteristics of signaling networks, and together cover the majority of examples in signal transduction pathways, modeled with mass action kinetics.

### Interferon signaling and differential activities

Type I interferons are a family of highly related proteins that regulate many different cellular functions (showing, among others, antiviral, antiproliferative and immunomodulatory activities). All Type I interferon subtypes (including IFN*α*2 and IFN*β*) interact with the same pair of receptor subunits at the cell membrane and induce the activation of the JAK/STAT pathway [38]. The different cellular responses (outputs) after IFN treatment depend on the IFN dose, receptor binding affinity (through a different stability of the ternary complex [39]) and the cell specific context (namely receptor density) [40]. However, the detailed cellular mechanisms translating these input and cell context differentials into specific biological response patterns are still unknown.

In this work we explore (sub)networks potentially playing a key role in the pathway dynamics, where the capacity for multiple steady states would reflect the outcome of a cell decision process. To this aim we search for bistability in different subnetwork structures compatible with current biological knowledge about the interferon signaling pathway. Specifically, we search at three different levels: ligand-receptor interactions at the cell membrane, early STAT signaling and STAT signaling including the expression of key proteins.

## Methods

### Basic notation and concepts

Firstly, we introduce the necessary concepts from the Chemical Reaction Network Theory formalism, the mathematical notation used throughout the paper, as well as the fundamental assumptions of the methods presented next.

#### Reaction network

A reaction network consists of *N* species participating in *R* reactions with given kinetics. In the *graph of complexes* of a reaction network (also referred to as *C*-graph), edges represent the reactions *r*_1_, …, *r_R_* and nodes are the sets of species at both sides of the reaction arrows, termed complexes $C_{1},\ldots ,\;C_{M}$. The number of nodes in the graph, i.e. the number of complexes of the network, is denoted by *M*.

In the context of cell signaling, basal or constitutive formation of a protein *A* is encoded as a pseudo reaction from a zero complex ∅ → *A*, while degradation of the protein is represented by a reaction to the zero complex *A* → ∅. The translocation of a species from/to another compartment is also represented by a pseudo reaction. For example, if a protein in the cytoplasm *A*_*cyt*_ is translocated into the nucleus the corresponding pseudo-reaction is *A*_*cyt*_ → *A*_*nuc*_.

**Fig 1:**
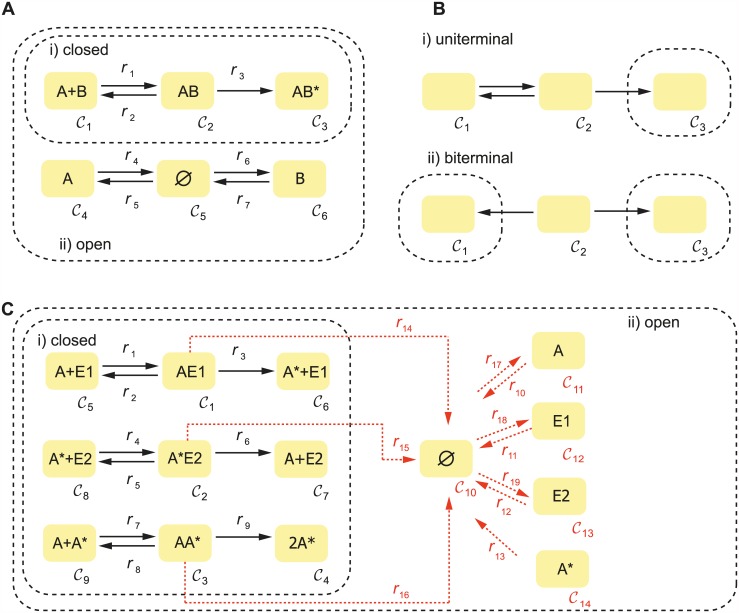
A) An example network where (i) protein *A* and protein *B* interact to form a complex which is further activated. The open version (ii) includes constitutive (basal) formation and degradation of both proteins *A* and *B* through pseudo reactions from/to the zero complex. B) An example for (i) uniterminal and (ii) biterminal networks. C) Signal transduction motif in its (i) closed version and (ii) semi-diffusive version in which the basal formation and degradation of *E*1, *A* and *E*2 is taken into account.

Let us consider the example in Fig 1A, where proteins *A* and *B* bind to form the complex *AB* which is further converted into its active form AB*. In addition, proteins *A* and *B* are being constitutively expressed (as indicated by pseudo reactions from the complex ∅), and degraded (as indicated by pseudo reactions to the complex ∅).

This reaction network contains *N* = 4 species with concentrations *c*_1_ = [*A*], *c*_2_ = [*B*], *c*_3_ = [*AB*] and *c*_4_ = [AB*] participating in *R* = 7 reactions, and its associated graph has *M* = 6 complexes.

#### Molecularity matrix

The molecularity matrix of a reaction network is an *N* × *M* matrix denoted by *Y* such that *Y*_*ij*_ is the molecularity of species *i* in complex *j*. The rank of the molecularity matrix is denoted by *ρ*. For the example network

$$\begin{array}{cc} & \begin{array}{cccccc} C_{1} & C_{2} & C_{3} & C_{4} & C_{5} & C_{6} \end{array}\\ \begin{array}{ccc} Y & = & \begin{array}{l} A\\ B\\ AB\\ AB^{*} \end{array} \end{array} & \left(\begin{array}{cccccc} 1 & 0 & 0 & 1 & 0 & 0\\ 1 & 0 & 0 & 0 & 0 & 1\\ 0 & 1 & 0 & 0 & 0 & 0\\ 0 & 0 & 1 & 0 & 0 & 0 \end{array}\right) \end{array}$$

and *ρ* = 4.

#### Stoichiometric matrix

The (species) stoichiometric matrix is an *N* × *R* matrix denoted by *S* such that:

$$\begin{array}{c} S_{ij}=\beta_{ij}-\alpha_{ij}, \end{array}$$

where *β*_*ij*_ is the molecularity of the species *i* in the set of products and *α*_*ij*_ is the molecularity of the species *i* in the set of educts of reaction *j*. For the example network:

$$\begin{array}{cc} & \begin{array}{ccccccc} r_{1} & r_{2} & r_{3} & r_{4} & r_{5} & r_{6} & r_{7} \end{array}\\ \begin{array}{ccc} S & = & \begin{array}{l} A\\ B\\ AB\\ AB^{*} \end{array} \end{array} & \left(\begin{array}{ccccccc} -1 & 1 & 0 & -1 & 1 & 0 & 0\\ -1 & 1 & 0 & 0 & 0 & 1 & -1\\ 1 & -1 & -1 & 0 & 0 & 0 & 0\\ 0 & 0 & 1 & 0 & 0 & 0 & 0 \end{array}\right). \end{array}$$

The stoichiometric matrix *S* can be computed from the molecularity matrix *Y* by taking into account that every reaction from complex *i* to complex *j* has an associated vector *Y*_*j*_−*Y*_*i*_ where *Y*_*j*_ and *Y*_*i*_ are respectively the *j*-th and *i*-th columns of the matrix *Y*. For example, the reaction vector associated with *r*_1_ is *Y*_2_−*Y*_1_ and the matrix *S* can be retrieved from the matrix *Y* as:

$$\begin{array}{c} S=\left[\begin{array}{ccccccc} Y_{2}-Y_{1}, & Y_{1}-Y_{2}, & Y_{3}-Y_{2}, & Y_{5}-Y_{4}, & Y_{4}-Y_{5}, & Y_{6}-Y_{5}, & Y_{5}-Y_{6} \end{array}\right]. \end{array}$$

The rank of the stoichiometric matrix is denoted by *s*.

#### Mass conservation relations

We consider systems that can exchange energy with the environment (thermodynamically non-isolated systems), and distinguish between open and closed systems based on whether or not they can exchange matter with the environment [41]. Closed networks, often called *conservative* networks in the CRNT literature [42, 43], do not exchange mass with the exterior. In open reaction networks, the exchange of matter with the environment can appear explicitly (via pseudo-reactions with the zero complex representing the environment) or not (like in reactions of the type *A* → 2*A*).

During a reaction mechanism, conservation relations (linear combinations of concentrations that remain constant during the process) might appear. Semi-positive conservation relations (positive linear combinations of species concentrations left invariant by the reactions), represent *conserved moieties*, i.e. biochemical units that participate without loss of integrity in a reaction mechanism [44–46]. Here we refer to these semi-positive conservation relations as mass conservation relations.

Let *B* be a *N* × *λ* matrix whose columns span the nullspace of *S*^*T*^, such that *S*^*T*^ *B* = 0. The matrix spanning the nullspace of *S*^*T*^ is not uniquely determined, and we choose *B* such that all its entries are non-negative (note that this is always possible provided that each conservation law represents conservation of a chemical unit or moiety). For the networks under study, each conservation relation corresponds to a conserved moiety (networks with other types of conservation relations are not in the scope of this paper). We denote by ${ℳ}_{1},\ldots ,\;{ℳ}_{\lambda }$ the conserved moieties of the network. The number of mass conservation relations is given by *λ* = *N* − *s* where *N* is the number of species and *s* is the rank of *S*.

Let us take a reference concentration vector *c*_0_, compute the vector of *mass conservation constants* *σ* = *B*^*T*^ *c*_0_, and define:

$$\begin{array}{c} W\left(c,\sigma \right)=B^{T}c-\sigma . \end{array}$$ (1)

The so called *reaction polyhedron* or *stoichiometric compatibility class* for the reference concentration vector (*c*_0_) consists of all *c* ≥ 0 such that

$$\begin{array}{c} W(c,\sigma )=0. \end{array}$$ (2)

A reaction network is closed if there is a strictly positive row vector *ϑ* with *N* elements such that *ϑS* = 0. A network is open if there is no such vector. Note that a network can be open and still have some mass conservation relations.

For the open system in Fig 1A, the rank of the stoichiometric matrix is *s* = 4. Since the number of species is also *N* = 4, we have that *λ* = 0 and, hence, there are no mass conservation laws. However, if we consider only its closed counterpart without the pseudo-reactions, the corresponding stoichiometric matrix reads:

$$\begin{array}{cc} & \begin{array}{ccc} r_{1} & r_{2} & r_{3} \end{array}\\ \begin{array}{ccc} S & = & \begin{array}{l} A\\ B\\ AB\\ AB^{*} \end{array} \end{array} & \left(\begin{array}{ccc} -1 & 1 & 0\\ -1 & 1 & 0\\ 1 & -1 & -1\\ 0 & 0 & 1 \end{array}\right) \end{array},$$

we have that *λ* = 2, and we obtain:

$$\begin{array}{cc} & \begin{array}{cccc} A & B & AB & AB^{*} \end{array}\\ \begin{array}{ccc} B^{T} & = & \begin{array}{l} {ℳ}_{1}\\ {ℳ}_{2} \end{array} \end{array} & \left(\begin{array}{cccc} 1 & 0 & 1 & 1\\ 0 & 1 & 1 & 1 \end{array}\right) \end{array},$$

implying that there are two mass conservation relations of the form:

$$\begin{array}{c} c_{1}+c_{3}+c_{4}=A_{0},\;\;\;\;\;c_{2}+c_{3}+c_{4}=B_{0}, \end{array}$$

where *A*_0_ = *c*_10_ + *c*_30_ + *c*_40_ and *B*_0_ = *c*_20_ + *c*_30_ + *c*_40_ (index 0 indicates initial amount) are the (constant) total amounts of species *A* and *B*, respectively, in all their forms. Note that the strictly positive vector *ϑ* = (1, 1, 2, 2) fulfills *ϑS* = 0, as it corresponds to a closed network.

Throughout, we assume that the dynamics of a reaction network are described by a set of ordinary differential equations (ODEs), which is commonly written in this compact form:

$$\begin{array}{c} \dot{c}=Sv\left(c,k\right) \end{array}$$ (3)

as the product of the stoichiometric matrix *S* and the vector of reaction rates *v*(*c*, *k*). Eq (3) together with an initial condition *c*_(*t* = 0)_ = *c*_0_ define an initial value problem.

#### Multistationarity in biochemical reaction networks

We say that a biochemical reaction network is multistationary if it has the capacity for multiple positive equilibria, i.e., if there exist *k* > 0, *c*_1_ > 0 and *c*_2_ > 0 with *c*_1_ ≠ *c*_2_ such that *Sv*(*c*_1_, *k*) = *Sv*(*c*_2_, *k*) = 0 in Eq (3) and *c*_1_, *c*_2_ belong to the same stoichiometric compatibility class (we use the notation *x* > 0 to indicate a strictly positive vector, i.e. all entries of *x* are strictly greater than zero). Note that, without mass conservation, the stoichiometric compatibility class of any positive equilibrium is the positive orthant.

In this work we provide methods to detect multi-steady state behavior arising through saddle-node bifurcations (see S1 Appendix for a detailed explanation on the implications of saddle-nodes and saddle-node bifurcations in multistationarity).

#### Mass action kinetics

A reaction network is not described completely until we define the kinetics of the participating reactions. In this work we consider mass action kinetics, for which the rates of the reactions are of the form:

$$\begin{array}{c} v_{j}=k_{j}\prod_{i=1}^{N}c_{i}^{\alpha_{ij}},\;\;\;\;j=1,\ldots ,R. \end{array}$$ (4)

Within this framework the number of parameters of the model, i.e. the number of kinetic rate constants *k*, is equal to the number of reactions *R*. Depending on the context, as we will see later, it might be more convenient to denote the reaction rates and kinetic constants using the labels of the source (educt) and product complexes of the reactions. Mass action law is frequently preferred in models of signaling pathways, since Michaelis-Menten and similar kinetics employ assumptions on time- and concentration-scale separation that are rarely fulfilled in signaling [47–49].

#### Main assumptions

In what follows, we consider only networks fulfilling the assumptions:

*A*.1 Every reaction is endowed with mass action kinetics. As indicated, this is a common assumption in signaling models, where every process can be ultimately represented by mass action kinetics.
*A*.2 The network admits a strictly positive steady state (where the concentrations of all the species are positive). This assumption is also mild in the context of signaling, due to the inherent reversibility of most protein-protein interactions. In practice, a signaling model can always be modified without loss of generality to avoid zero steady states (assuming very low concentrations instead) such that the assumption is fulfilled.

These assumptions are common to both methods presented in this paper.

### A method to detect multistationarity in signaling networks with mass conservation

In this section we present a deficiency oriented approach to efficiently detect multi-steady state behavior in signaling pathways with mass conservation (this includes closed networks and open networks with mass conservation). Based on a previous work [37] we here develop sufficient conditions for the existence of a saddle-node in the specific context of signaling pathways. We formulate the search as an optimization problem that provides, in case it is detected, the coordinates of a saddle-node. From this point, a continuation of equilibrium is started by means of a standard continuation algorithm, to verify whether the saddle-node is indeed a saddle-node bifurcation (which leads to two different equilibrium branches).

#### Additional assumptions

This (*deficiency*-oriented) approach is applicable to any cell signaling network fulfilling *A*.1 and *A*.2 under an extra mild assumption concerning the graph of complexes, namely, the so-called *linkage classes* of the network.

The graph of complexes for a reaction network contains ℓ disconnected subgraphs, also called linkage classes ${ℒ}_{1},\ldots ,{ℒ}_{\ell }$.

Two nodes $C_{i},\;C_{j}$, are said to be *strongly linked* if there is a directed path from $C_{i}$ to $C_{j}$ and also a directed path from $C_{j}$ to $C_{i}$. A *terminal strong linkage class* is a maximal set of nodes within a disconnected subgraph such that there is no edge pointing to any other set of nodes that are strongly linked.

We say that a network graph (or for simplicity, a network) is *uniterminal* if every linkage class in the graph contains only one terminal strong linkage class. For illustrative purposes we depict in Fig 1B a linkage class (i) which contains one terminal strong linkage class (and, therefore, the network is uniterminal), and a linkage class (ii) which contains two terminal strong linkage classes (biterminal). In order to apply the deficiency-oriented approach, we need the following assumption to be fulfilled:

*A*.3 The network graph is uniterminal.

Networks which are non-uniterminal are considered, in general, to be unsuited for the description of real chemical systems [21]. In the context of cell signaling it can be said that the vast majority of the networks are uniterminal.

To illustrate this approach let us consider the signal transduction mechanism presented in Fig 1C (i). The subgraph containing nodes $C_{1},\ldots ,\;C_{9}$ is a closed network in which protein *A* is transformed into its active form A* by enzyme *E*1, and deactivated by enzyme *E*2. Furthermore, protein *A* activates itself through the formation of an intermediate complex AA*. The concentrations of the species involved are *c*_1_ = [*A*], *c*_2_ = [*E*1], *c*_3_ = [*E*2], *c*_4_ = [A*], *c*_5_ = [*AE*1], *c*_6_ = [A*E2] and *c*_7_ = [AA*].

#### Labeling of the complexes and reactions

We label the complexes with numbers from 1 to *M* (the labeling is arbitrary) before constructing the matrices of the network. In the example, once we label the complexes $C_{1},\ldots ,\;C_{9}$ as in Fig 1C, the molecularity matrix is:

$$\begin{array}{cc} & \begin{array}{ccccccccc} C_{1} & C_{2} & C_{3} & C_{4} & C_{5} & C_{6} & C_{7} & C_{8} & C_{9} \end{array}\\ \begin{array}{ccc} Y & = & \begin{array}{l} A\\ E1\\ E2\\ A^{*}\\ AE1\\ A^{*}E2\\ AA^{*} \end{array} \end{array} & \left(\begin{array}{ccccccccc} 0 & 0 & 0 & 0 & 1 & 0 & 1 & 0 & 1\\ 0 & 0 & 0 & 0 & 1 & 1 & 0 & 0 & 0\\ 0 & 0 & 0 & 0 & 0 & 0 & 1 & 1 & 0\\ 0 & 0 & 0 & 2 & 0 & 1 & 0 & 1 & 1\\ 1 & 0 & 0 & 0 & 0 & 0 & 0 & 0 & 0\\ 0 & 1 & 0 & 0 & 0 & 0 & 0 & 0 & 0\\ 0 & 0 & 1 & 0 & 0 & 0 & 0 & 0 & 0 \end{array}\right) \end{array},$$

and the stoichiometric matrix reads:

$$\begin{array}{c} S=\left[Y_{1}-Y_{5},Y_{5}-Y_{1},Y_{6}-Y_{1},Y_{2}-Y_{8},Y_{8}-Y_{2},Y_{7}-Y_{2},Y_{3}-Y_{9},Y_{9}-Y_{3},Y_{4}-Y_{3}\right]. \end{array}$$ (5)

Let us introduce a matrix *Λ* in which entry (*i*, *j*) corresponds to node $C_{i}$ in linkage class $L_{j}$, such that:

$$\Lambda_{i,j}=\left\{\begin{array}{cc} 1 & \;\text{if}\;C_{i}\in L_{j}\\ 0 & \;\text{otherwise.} \end{array}\right.$$

The dimensions of the matrix *Λ* are *M* × *ℓ*. In the example (considering only the subgraph corresponding to the closed system) there are *M* = 9 nodes distributed among *ℓ* = 3 linkage classes such that:

$$\begin{array}{cc} & \begin{array}{ccccccccc} C_{1} & C_{2} & C_{3} & C_{4} & C_{5} & C_{6} & C_{7} & C_{8} & C_{9} \end{array}\\ \begin{array}{ccc} \Lambda^{T} & = & \begin{array}{l} {ℒ}_{1}\\ {ℒ}_{2}\\ {ℒ}_{3} \end{array} \end{array} & \left(\begin{array}{ccccccccc} 1 & 0 & 0 & 0 & 1 & 1 & 0 & 0 & 0\\ 0 & 1 & 0 & 0 & 0 & 0 & 1 & 1 & 0\\ 0 & 0 & 1 & 1 & 0 & 0 & 0 & 0 & 1 \end{array}\right) \end{array}.$$

Within the framework of the deficiency-oriented approach, as indicated in the previous section (**Mass action kinetics**) it is more convenient to denote the reaction rates according to the educt and product complexes, such that the kinetic constant of the reaction from complex *j* to complex *k* is denoted as *k*_*jk*_. In this way, each complex has an associated mass action monomial of the form:

$$\begin{array}{c} \psi_{j}\left(c\right)=\prod_{i=1}^{N}c_{i}^{y_{ij}}. \end{array}$$ (6)

We can define a vector of monomials *ψ*(*c*) = (*ψ*_1_, …, *ψ_M_*)^T^. The relationship between the vector containing the reaction rates as defined in Eq (4) and the vector of monomials is given by:

$$\begin{array}{c} v(c,k)=diag(k)U\psi (c), \end{array}$$

where *diag*(*k*) is an *R*-diagonal matrix containing the kinetic constants of the reactions and *U* ∈ {0, 1}^*R* × *M*^ is a matrix that *selects* the source complexes of the reactions [50].

The vector of mass action monomials *ψ*(*c*) for the example network reads:

$$\begin{array}{c} \psi \left(c\right)={\left(\begin{array}{ccccccccc} c_{5}, & c_{6}, & c_{7}, & c_{4}^{2}, & c_{1}c_{2}, & c_{2}c_{4}, & c_{1}c_{3}, & c_{3}c_{4}, & c_{1}c_{4} \end{array}\right)}^{T}. \end{array}$$

#### Model equations

Within this framework the dynamics Eq (3) of a reaction network can be encoded in an equivalent set of ODEs of the form:

$$\begin{array}{c} \dot{c}=YA\psi \left(c\right), \end{array}$$ (7)

where *Y* is the molecularity matrix, *A* contains the kinetic rate constants and *ψ*(*c*) is the vector of mass action monomials Eq (6). Matrix *A* is built such that its diagonal elements *A*_*i*, *i*_ contain the negative of the sum of the kinetic rate constants corresponding to the reactions going out of complex $C_{i}$, while the off-diagonal elements *A*_*i*, *j*_ contain the kinetic constants of the reactions going from complex $C_{i}$ to complex $C_{j}$. In the example:

$$\begin{array}{c} A=\left(\begin{array}{ccccccccc} -(k_{15}+k_{16}) & 0 & 0 & 0 & k_{51} & 0 & 0 & 0 & 0\\ 0 & -(k_{27}+k_{28}) & 0 & 0 & 0 & 0 & 0 & k_{82} & 0\\ 0 & 0 & -(k_{34}+k_{39}) & 0 & 0 & 0 & 0 & 0 & k_{93}\\ 0 & 0 & k_{34} & 0 & 0 & 0 & 0 & 0 & 0\\ k_{15} & 0 & 0 & 0 & -k_{51} & 0 & 0 & 0 & 0\\ k_{16} & 0 & 0 & 0 & 0 & 0 & 0 & 0 & 0\\ 0 & k_{27} & 0 & 0 & 0 & 0 & 0 & 0 & 0\\ 0 & k_{28} & 0 & 0 & 0 & 0 & 0 & -k_{82} & 0\\ 0 & 0 & k_{39} & 0 & 0 & 0 & 0 & 0 & -k_{93} \end{array}\right). \end{array}$$

Matrix *B* in Eq (1) can be computed from the stoichiometric matrix or, equivalently for the networks under study, from matrix *YA* such that (*YA*)^*T*^ *B* = 0, obtaining:

$$\begin{array}{cc} & \begin{array}{ccccccc} A & E1 & E2 & A^{*} & AE1 & A^{*}E2 & AA^{*} \end{array}\\ \begin{array}{ccc} B^{T} & = & \begin{array}{l} M_{1}\\ M_{2}\\ M_{3} \end{array} \end{array} & \left(\begin{array}{ccccccc} 0 & 1 & 0 & 0 & 1 & 0 & 0\\ 0 & 0 & 1 & 0 & 0 & 1 & 0\\ 1 & 0 & 0 & 1 & 1 & 1 & 2 \end{array}\right) \end{array}.$$

Therefore, we get the following conservation laws:

$$\begin{array}{c} c_{2}+c_{5}=E1_{0},\;\;\;\;\;c_{3}+c_{6}=E2_{0},\;\;\;\;\;c_{1}+c_{4}+c_{5}+c_{6}+2c_{7}=A_{0}, \end{array}$$

where *A*_0_, *E*1_0_ and *E*2_0_ are constants which represent, respectively, the total amounts of the species *A*, *E*1 and *E*2, including all their forms. Note that the strictly positive vector *ϑ* = (1, 1, 1, 1, 2, 2, 2) fulfills *ϑS* = 0, as it corresponds to a closed network.

#### Deficiency and equilibrium manifold

The *deficiency* of a network is a nonnegative integer number defined as:

$$\begin{array}{c} \delta =M-\ell -s. \end{array}$$

According to the Deficiency Zero Theorem [21], if the deficiency of a reaction network is zero and, in addition, the network is weakly reversible (every linkage class is a strong terminal linkage class), then, regardless of the values of the kinetic constants, each stoichiometric compatibility class contains precisely one unique steady state, and this steady state is asymptotically stable. Next we obtain an expression for the locus of equilibria of a reaction network in terms of what we call *deficiency parameters*.

For the uniterminal networks under study, the deficiency *δ* coincides with the dimension of the so-called *deficiency subspace* D:= *Ker(Y)* ∩ *Im(A)*. A basis *ω*_1_, …, *ω_δ_* for the deficiency subspace can be computed following [37] and taking into account that:

$$\begin{array}{c} span\left(\omega \right)=Ker\left[\begin{array}{c} Y\\ \Lambda^{T} \end{array}\right]. \end{array}$$ (8)

For the example network a basis is:

$$\begin{array}{l} \omega_{1}={\left(\begin{array}{ccccccccc} 0 & 0 & 0 & 0 & 1 & -1 & -1 & 1 & 0 \end{array}\right)}^{T},\\ \omega_{2}={\left(\begin{array}{ccccccccc} 0 & 0 & 0 & -1 & -1 & 1 & 0 & 0 & 1 \end{array}\right)}^{T}. \end{array}$$

Since the vector *Aψ* is a linear combination of the vectors of the basis of D, we have:

$$\begin{array}{c} A\psi =\sum_{i=1}^{\delta }\alpha_{i}\omega_{i}, \end{array}$$ (9)

which contains *M* − *ℓ* linearly independent algebraic equations. We refer to the linear coefficients *α*_1_, …, *α_δ_* as *deficiency parameters*. Substituting the vector *ψ* by the corresponding mass action monomials, we obtain a set of *M* − *ℓ* linearly independent equations in terms of the state vector *c*, the deficiency parameter vector *α* and the kinetic parameter vector *k*, namely:

$$\begin{array}{c} H(c,\alpha ,k)=0, \end{array}$$ (10)

which describes the locus of equilibria of Eq (7). Note that, for fixed *k*, the dimension of the manifold described by Eq (10) is *λ*, coinciding with the number of mass conservation laws in the system (since there are *N* + *δ* variables and *M* − *ℓ* equations, and *λ* = *N* − *s*). The set of equations:

$$\begin{array}{l} H\left(c,\alpha ,k\right)=0\\ W\left(c,\sigma \right)=0 \end{array}$$ (11)

where *W* is given by Eq (1) describes the locus of equilibrium points compatible with the reaction polyhedron fixed by *σ*. Note that, for fixed *k* and *σ*, the system is square with *N*+*δ* variables and *N*+*δ* equations.

In the example, solving Eq (9) we obtain:

$$\begin{array}{rrr} k_{15}\psi_{1}-k_{51}\psi_{5} & = & \alpha_{1}-\alpha_{2}\\ k_{16}\psi_{1} & = & -\alpha_{1}+\alpha_{2}\\ k_{27}\psi_{2} & = & -\alpha_{1}\\ k_{28}\psi_{2}-k_{82}\psi_{8} & = & \alpha_{1}\\ k_{34}\psi_{3} & = & -\alpha_{2}\\ k_{39}\psi_{3}-k_{93}\psi_{9} & = & \alpha_{2}. \end{array}$$

Expressing the mass action monomials through the state variables we get the following equations for the equilibrium manifold Eq (10):

$$\begin{array}{rrr} c_{1}c_{2}-k_{15}/k_{51}c_{5}+\alpha_{1}/k_{51}-\alpha_{2}/k_{51} & = & 0\\ c_{5}+\alpha_{1}/k_{16}-\alpha_{2}/k_{16} & = & 0\\ c_{6}+\alpha_{1}/k_{27} & = & 0\\ c_{3}c_{4}-k_{28}/k_{82}c_{6}+\alpha_{1}/k_{82} & = & 0\\ c_{7}+\alpha_{2}/k_{34} & = & 0\\ c_{1}c_{4}-k_{39}/k_{93}c_{7}+\alpha_{2}/k_{93} & = & 0. \end{array}$$ (12)

According to the deficiency and the dimension of the equilibrium manifold, we classify the networks into three groups [51]:

*G*.1 *Proper networks* where *N* = *M* − ℓ and, therefore, *λ* = *δ*.
*G*.2 *Over-dimensioned networks*, where *N* < *M* − ℓ and, therefore, *λ* < *δ*.
*G*.3 *Under-dimensioned networks*, where *N* > *M* − ℓ and, therefore, *λ* > *δ*.

For proper networks, once we fix the values of the deficiency parameters *α*, the equilibrium point is fixed. For over-dimensioned networks, once we fix the values of *λ* components of the deficiency parameter vector *α*, the equilibrium point is fixed. Finally, for under-dimensioned networks, we need to fix *α* and *λ* − *δ* components of the state vector such that the equilibrium point is fixed.

The network of the example is under-dimensioned since the dimension of the manifold is *λ* = 3 and the deficiency is *δ* = 2. As it can be deduced from Eq (12), as soon as we fix the values of *α*_1_, *α*_2_ and *c*_2_ the equilibrium point is fixed.

#### Sufficient conditions for a saddle-node in presence of mass conservation

Once we obtain the equations of the equilibrium manifold and the reaction polyhedron we define the following matrix (in what follows *D*_*x*_ *F* denotes the Jacobian of *F* with respect to *x*):

$$\begin{array}{c} G\left(c,\alpha ,k\right)=\left(\begin{array}{cc} D_{c}H & D_{\alpha }H\\ D_{c}W & D_{\alpha }W \end{array}\right), \end{array}$$ (13)

where *D*_*c*_ *W* = *B*^*T*^ and *D*_*α*_ *W* = 0. Here it is important to remark that this matrix is always square, of dimensions *N* + *δ*, for arbitrary deficiency and manifold dimensions. In the example we obtain a 9 × 9 matrix:

$$\begin{array}{c} G=\left(\begin{array}{ccccccccc} c_{2} & c_{1} & 0 & 0 & -k_{15}/k_{51} & 0 & 0 & 1/k_{51} & -1/k_{51}\\ 0 & 0 & 0 & 0 & 1 & 0 & 0 & 1/k_{16} & -1/k_{16}\\ 0 & 0 & 0 & 0 & 0 & 1 & 0 & 1/k_{27} & 0\\ 0 & 0 & c_{4} & c_{3} & 0 & -k_{28}/k_{82} & 0 & 1/k_{82} & 0\\ 0 & 0 & 0 & 0 & 0 & 0 & 1 & 0 & 1/k_{34}\\ c_{4} & 0 & 0 & c_{1} & 0 & 0 & -k_{39}/k_{93} & 0 & 1/k_{93}\\ 0 & 1 & 0 & 0 & 1 & 0 & 0 & 0 & 0\\ 0 & 0 & 1 & 0 & 0 & 1 & 0 & 0 & 0\\ 1 & 0 & 0 & 1 & 1 & 1 & 2 & 0 & 0 \end{array}\right). \end{array}$$

In presence of mass conservation, if there exist *c* > 0, *k* > 0 and *α* such that:

*C*.1 $$\begin{array}{c} H(c,\alpha ,k)=0, \end{array}$$
*C*.2 $$\begin{array}{c} D_{c}H\left(c,\alpha ,k\right)\;\;\text{is of full rank}, \end{array}$$
*C*.3 $$\begin{array}{c} \text{rank}(G(c,\alpha ,k))=N+\delta -1, \end{array}$$

the system Eq (11) has a limit point or saddle-node at the (strictly positive) equilibrium given by *c*, *k*. The proof is included in S1 Appendix.

#### Effective search for multistationarity

Next we propose a two step algorithm for an efficient and systematic search of multistationarity based on the deficiency criterion. First, we search for the kinetic rate constants and steady state concentrations such that the conditions for a saddle-node (*C*.1 to *C*.3) are met. We define the decision vector x for the optimization problem as:

x = (*k*_1_, …, *k_R_*, *α*_1_, …, *α_λ_*) in case of proper and over-dimensioned networks
x = (*k*_1_, …, *k_R_*, *α*_1_, …, *α_δ_*, *c*_1_, …, *c_λ − δ_*) in case of under-dimensioned networks.

The objective function to be minimized (under appropriate constraints) is:

$$\begin{array}{c} F_{def}\left(x\right)=det{\left(G\left(c,\alpha ,k\right)\right)}^{2} \end{array}$$

such that, at the optimum *F*_*def*_(x^*opt*^) = 0 the conditions for a saddle-node (*C*.1 to *C*.3) are fulfilled. The optimization problem can be formulated as:

$$\begin{array}{ll} \text{Minimize} & F_{def}\left(\text{x}\right)\\ \text{subject to:} & H\left(c,\alpha ,k\right)=0,\\ & \text{rank}\left(G\left(c,\alpha ,k\right)\right)=N+\delta -1,\\ & \text{rank}\left(D_{c}H\left(c,\alpha ,k\right)\right)=min\left(N,M-l\right),\;\\ & c\;>\;0\;c\in {\mathbb{R}}^{N},\\ & {\text{x}}_{L}\;\leq \text{ x }\leq \;{\text{x}}_{U}\;\left({\text{x}}_{L},{\text{x}}_{U}\in {\mathbb{R}}^{R+\lambda }\right). \end{array}$$ (14)

The lower and upper bounds for the decision variables are denoted by x_*L*_ and x_*U*_, respectively, and the constraint *c* > 0 is imposed according to assumption *A*.2. Note that decision variables corresponding to kinetic rate constants have positive lower bounds, while decision variables corresponding to deficiency parameters might take negative values. This optimization problem (14) is non-convex and potentially multi-modal. Consequently, a global optimization algorithm [52] has to be used to search for the optimal solution. Here it is important to remark that, if the network is multistationary, the problem has infinitely many solutions for which *F*_*def*_(x) = 0.

Finally, in order to verify whether the saddle-node is a saddle-node bifurcation, we start a continuation of equilibrium in forward and backward directions by numerical continuation [53]. In case the saddle node is a saddle node bifurcation, two branches of equilibria emerge (there is multistationarity).

**Fig 2:**
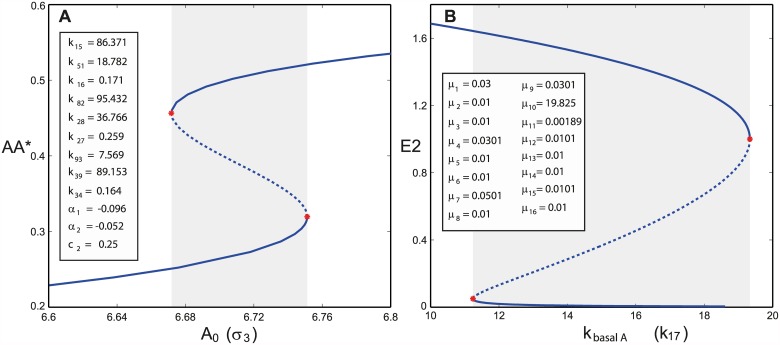
Bifurcation diagrams corresponding to the example network in Fig 1C in its A) closed and B) open versions. Tangent bifurcations are indicated by red stars. Stable and unstable equilibria branches are indicated by solid and dashed lines, respectively. Bistability regions are enclosed within the grey rectangles.

An optimal solution for the example network is found for the set of parameters in Fig 2A. The computation time for the optimization with global scatter search [52] was 40 seconds on an Intel 2.8 GHz Xeon processor. Taken with these parameters, the network is bistable as shown in the bifurcation diagram, where the steady state concentration of the species AA* is plotted against the mass conservation constant *σ*_3_ (also denoted as *A*_0_), which is taken as the bifurcation parameter. The continuation analysis is performed using Cl-Matcont [53].

### A method to detect multistationarity in signaling networks without mass conservation

In this section we present an injectivity-oriented approach to efficiently detect multistationary behavior in signaling pathways in absence of mass conservation relations. The approach is based on previous work by Craciun and Feinberg [23] in the context of Continuous Flow Stirred Tank Reactors (CFSTR). There, the authors propose sufficient conditions for multistationarity in fully diffusive networks (where all the species are present in the feed and outflow streams). Here, we first define the class of *semi-diffusive* networks, which complies with the natural assumptions for signaling pathways, and we develop sufficient conditions for a saddle-node to occur. We propose an algorithm to efficiently search for parameters that satisfy the saddle-node conditions in terms of reaction fluxes, and show how to retrieve the exact coordinates (in terms of kinetic rate constants and steady state concentrations) of the saddle-node, starting from the set of optimal fluxes. Finally, a continuation of equilibrium is started in forward and backward directions providing, in case that the saddle-node is a saddle-node bifurcation, two branches of equilibria.

#### Additional assumptions

This approach is applicable to any cell signaling network fulfilling *A*.1 and *A*.2 under an additional assumption ensuring that there is no mass conservation, to be introduced next.

In the original work [23] the absence of mass conservation is guaranteed by considering *fully diffusive* systems, i.e. systems with input and output flows for every species. In the context of cell signaling this would entail degradation and basal formation of all the species. This assumption does not fit well with signaling pathways, where, for example, single proteins but not signaling complexes are subject to basal formation.

In order to cope with realistic cell signaling models we introduce here what we call *semi-diffusive* networks. Let us first adopt the notation by [23] and differentiate among *true* reactions (belonging to the biochemical reaction mechanism), *outflow* reactions (degradation or flow out of the cell compartment) and *feed* reactions (basal formation or flow into the cell compartment). In Structural Analysis *true* reactions are referred to as *internal* reactions and feed and outflow reactions as *exchange* reactions [50].

First we calculate the conservation laws of the subsystem consisting only of the true reactions. In *semi-diffusive* networks, there is degradation of all species, as well as inflow (or basal formation) of one species per conservation law. This species is chosen to be the free form of the corresponding protein participating in the conservation law. If the network is not weakly reversible, we need to ensure that our choice is compatible with *A*.2. The species in the inflow, i.e., the species which are being constitutively generated, are denoted as *key* species. Now, we are ready to state the assumption that (together with *A*.1 and *A*.2) must be verified:

*A*.4 The reaction network is semi-diffusive.

Importantly, cell signaling pathways usually fit into the category of *semi-diffusive* networks.

The open network in Fig 1C (ii) is an example of a semi-diffusive network with true reactions *r*_1_, …, *r*_9_, outflow reactions *r*_10_, …, *r*_16_ corresponding to degradation of each of the species and inflow reactions *r*_17_, …, *r*_19_ corresponding to basal formation of the *key* species *A*, *E*1 and *E*2 (one per conservation law).

#### Labeling of the reactions

In this approach we enumerate the kinetic rate constants according to the labels of the corresponding reactions. The rate of reaction *r*_*j*_ is denoted by *v*_*j*_ and its corresponding kinetic rate constant by *k*_*j*_. Thereby, in the example the kinetic constant of the reaction going from complex $C_{5}$ to complex $C_{1}$ is denoted in what follows by *k*_1_, since it corresponds to reaction *r*_1_. The reaction rate or flux is denoted by *v*_1_.

#### Model equations

The model for the cell signaling pathway can be encoded in a system of ODEs of the form:

$$\begin{array}{c} \dot{c}=K+S_{to}v_{to}\left(c,k\right), \end{array}$$ (15)

where *K* ≥ 0 is the constant inflow term while *S*_*to*_ and *v*_*to*_(*c*, *k*) are the stoichiometric matrix and vector of reaction rates containing only the true and outflow reactions. We build the molecularity matrix *Y*_*to*_ as follows: let *Y* be the molecularity matrix of the true reactions subsystem (i.e. the subsystem consisting only of the true reactions). First we add to the matrix *Y* a column corresponding to the zero complex, and then we add the columns corresponding to the species that, being in the outflow, do not appear alone in a complex of the true reactions subsystem. For the example in Fig 1C, the species *A*, *E*1, *E*2 and A* do not appear alone in a complex participating in a true reaction, and the molecularity matrix of the semi-diffusive network reads:

$$\begin{array}{c} Y_{to}=\left(\begin{array}{ccccccccc} 0 & 0 & 0 & 0 & 1 & 0 & 1 & 0 & 1\\ 0 & 0 & 0 & 0 & 1 & 1 & 0 & 0 & 0\\ 0 & 0 & 0 & 0 & 0 & 0 & 1 & 1 & 0\\ 0 & 0 & 0 & 2 & 0 & 1 & 0 & 1 & 1\\ 1 & 0 & 0 & 0 & 0 & 0 & 0 & 0 & 0\\ 0 & 1 & 0 & 0 & 0 & 0 & 0 & 0 & 0\\ 0 & 0 & 1 & 0 & 0 & 0 & 0 & 0 & 0 \end{array}\;|\;\begin{array}{ccccc} 0 & 1 & 0 & 0 & 0\\ 0 & 0 & 1 & 0 & 0\\ 0 & 0 & 0 & 1 & 0\\ 0 & 0 & 0 & 0 & 1\\ 0 & 0 & 0 & 0 & 0\\ 0 & 0 & 0 & 0 & 0\\ 0 & 0 & 0 & 0 & 0 \end{array}\right). \end{array}$$

Note that the tenth column (to the right of the vertical line) corresponds to the environment node.

The stoichiometric matrix *S*_*to*_ is built from the columns of the molecularity matrix, incorporating only the true and outflow reactions, i.e. *r*_1_, …, *r*_16_. Taking into account the stoichiometric matrix for the true reactions given by Eq (5), we can write the stoichiometric matrix including also the outflow reactions *S*_*to*_ as:

$$\begin{array}{c} S_{to}=\left[\begin{array}{c} S \end{array}\;|\;\begin{array}{c} -I_{N\times N} \end{array}\right], \end{array}$$

where *I*_*N* × *N*_ is the identity matrix containing the source complexes for every species in the network (in this case *N* = 7).

The inflow vector *K* contains the rates of basal formation for *A*, *E*1 and *E*2:

$$\begin{array}{c} K={\left(\begin{array}{ccccccc} v_{17} & v_{18} & v_{19} & 0 & 0 & 0 & 0 \end{array}\right)}^{T}, \end{array}$$

where *v*_17_ = *k*_17_, *v*_18_ = *k*_18_ and *v*_19_ = *k*_19_ are the (constant) reaction rates of *r*_17_, *r*_18_ and *r*_19_.

Let us build a matrix *Y*_*r*_ with columns corresponding to the source complexes for the true and outflow reactions, namely:

$$\begin{array}{c} Y_{r}=\left[\begin{array}{ccccccccc} Y_{5} & Y_{1} & Y_{1} & Y_{8} & Y_{2} & Y_{2} & Y_{9} & Y_{3} & Y_{3} \end{array}\;|\;\begin{array}{c} I_{7\times 7} \end{array}\right]. \end{array}$$

#### Jacobian and the injectivity property

When we denote by *f*(*c*, *k*) the right hand side of Eq (15), at equilibrium we have:

$$\begin{array}{c} f\left(c,k\right)=K+S_{to}v_{to}\left(c,k\right)=0. \end{array}$$ (16)

The inflow term *K* is a constant vector, and the Jacobian of the system reads:

$$\begin{array}{c} D_{c}f=S_{to}V_{to}\left(c,k\right),\;\;\;V_{to}\left(c,k\right)=\frac{\partial v_{to}\left(c,k\right)}{\partial c}. \end{array}$$

Since we assume mass action kinetics, the Jacobian can be easily computed from the network matrices as:

$$\begin{array}{c} D_{c}f=S_{to}diag\left(v_{to}\right)Y_{r}^{T}diag\left(c^{-1}\right). \end{array}$$

For a strictly positive steady state concentration *c* > 0 we define:

$$\begin{array}{c} p\left(c,k\right)=-S_{to}v_{to}\left(c,k\right), \end{array}$$ (17)

and at steady state we have:

$$\begin{array}{c} K=p(c,k). \end{array}$$

A network is said to be *injective* if *p*(*c*_1_, *k*) = *p*(*c*_2_, *k*) ⇒ *c*_1_ = *c*_2_ for all *k* > 0. If, on the contrary, there exist *c*_1_ > 0, *c*_2_ > 0 and *k* > 0 with *c*_1_ ≠ *c*_2_ such that *p*(*c*_1_, *k*) = *p*(*c*_2_, *k*), the network is not injective. It has been demonstrated by [23] that a network is injective if and only if:

$$\begin{array}{c} detD_{c}f\left(c,k\right)\neq 0,\;\;\text{for all}\;c>0,k>0. \end{array}$$

#### Sufficient conditions for a saddle-node in absence of mass conservation

For a *fully-diffusive* network, where all species are present in the inflow and outflow, if there exist *c* > 0, *k* > 0 such that:

C.4 $$\begin{array}{c} detD_{c}f\left(c,k\right)=0, \end{array}$$
C.5 $$\begin{array}{c} p(c,k)>0. \end{array}$$

the network is multistationary [23]. Importantly, non-injectivity (*C*.4) alone is not sufficient to ensure multiple positive steady states. The proof can be found in the original paper by Craciun and Feinberg [23].

For a *semi-diffusive* network, if there are *k* > 0, *c* > 0 such that:

C.6 $$\begin{array}{c} \text{rank}\left(D_{c}f\left(c,k\right)\right)=N-1, \end{array}$$
C.7 $$\begin{array}{l} p_{i}\left(c,k\right)>0\text{ if }c_{i}\text{ is a key species},\\ p_{i}\left(c,k\right)=0\text{ if }c_{i}\text{ is not a key species}. \end{array}$$

the system Eq (16) has a saddle-node at the (strictly positive) equilibrium given by *c*, *k*. The proof can be found in S1 Appendix.

#### Effective search for multistationarity

Next we propose an algorithm for an easy and systematic detection of multistationarity based on the injectivity criterion. First we formulate an optimization problem to detect a saddle-node. For convenience, we consider the fluxes (steady state reaction rates, as defined in Structural Analysis [44]), as decision variables. Let us denote by *μ* the vector containing the fluxes of the true and outflow reactions and compute:

$$\begin{array}{c} \bar{J}\left(\mu \right)=S_{to}diag\left(\mu \right)Y_{r}^{T}. \end{array}$$

We consider the following objective function in terms of the vector of fluxes *μ*:

$$\begin{array}{c} F_{inj}\left(\mu \right)=det{\left(\bar{J}\left(\mu \right)\right)}^{2}. \end{array}$$

Redefining expression (17) in terms of the vector of fluxes:

$$\begin{array}{c} \bar{p}\left(\mu \right)=-S_{to}\mu , \end{array}$$

the optimization problem can be formulated as:

$$\begin{array}{ll} \text{Minimize} & F_{inj}\left(\mu \right)\\ \text{subject to:} & {\bar{p}}_{i}\left(\mu \right)\geq 0\text{ if }c_{i}\text{ is a key species},\\ & {\bar{p}}_{i}\left(\mu \right)=0\text{ if }c_{i}\text{ is not a key species},\\ & \text{rank}\left(\bar{J}\left(\mu \right)\right)=N-1,\\ & \mu_{L}\;\leq \;\mu \;\leq \;\mu_{U}\;\left(\mu_{\left\{L,U\right\}}\in {\mathbb{R}}_{>0}^{R_{to}}\right), \end{array}$$ (18)

where the vector of fluxes *μ* is the decision vector and the lower and upper bounds for the decision variables are denoted by *μ*_*L*_ and *μ*_*U*_, respectively. Note that *μ*_*L*_ > 0 is imposed according to assumption *A*.2. At the optimum *F*_*inj*_(*μ*^*opt*^) = 0, the conditions *C*.6 and *C*.7 for a saddle-node are fulfilled.

This optimization problem (18) is potentially multi-modal and consequently a global optimization algorithm [52] is used to search for the solution.

If a vector *μ*^*opt*^ is found such that *F*_*inj*_(*μ*^*opt*^) = 0, we can retrieve the exact coordinates of a saddle-node in terms of the concentration vector and kinetic parameters. An equilibrium continuation (in forward and backward directions) is started from this point to analyze the behavior of the equilibrium curve in the vicinity of the saddle-node. If the saddle-node is also a saddle-node bifurcation (turning point), multistationarity appears.

The algorithm finds an optimal vector of fluxes *μ*^*opt*^ for our example network in Fig 1C such that *F*_*inj*_(*μ*^*opt*^) = 0 (values provided in the legend) and, therefore, the network has a saddle-node. The computation time for the optimization with global scatter search [52] was 80 seconds on an Intel 2.8 GHz Xeon processor. Here we set the steady state concentrations to 1, such that *k*_*i*_ = *μ*_*i*_ for *i* = 1, …, 16 and *k*_17_ = *p*_1_, *k*_18_ = *p*_2_, *k*_19_ = *p*_3_. In this way, the point *c* = 1 is a limit point bifurcation where a real eigenvalue crosses the imaginary axis. Starting from this point we compute the bifurcation diagram with respect to *k*_17_, which is the kinetic rate constant corresponding to basal formation of protein *A*. As it is shown in Fig 2B, there is a region of bistability enclosed by two limit point bifurcations.

## Results

Type I interferons regulate many different cellular functions showing, amongst others, antiviral, antiproliferative and immunomodulatory activities, depending on variables including the interferon concentration, the affinity towards the IFN receptor, or the cell receptor density. One of our goals during the modeling process of the interferon signaling pathway is to identify key regulators responsible for the differential cellular outcomes in response to IFN treatment, especially with respect to differential anti-proliferative and anti-viral activities.

**Fig 3:**
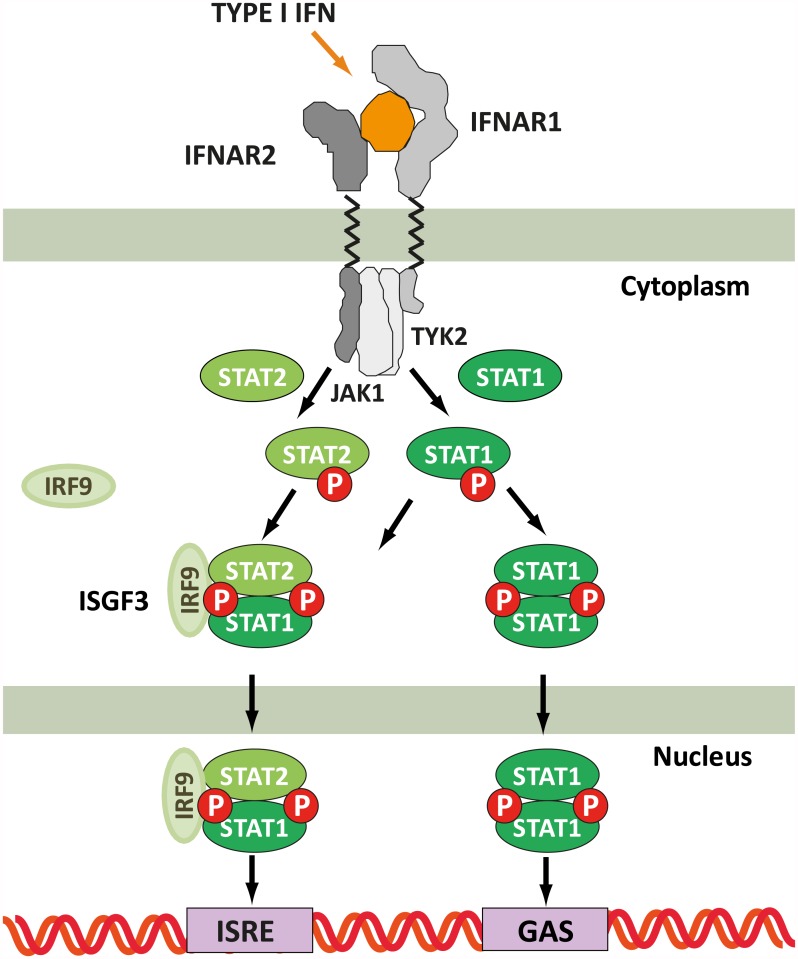
Simplified scheme of the interferon signaling pathway with scope from ligand binding to transcription of ISG genes (adapted from [55]). Type I IFNs can induce expression of genes with ISRE and GAS elements.

In Fig 3 we show a simplified scheme of the interferon signaling pathway, incorporating reactions from the ligand binding level to the transcriptional level. Interferon binds to the interferon receptor subunits IFNAR1 and IFNAR2 on the cell membrane to form a ternary complex that activates the JAK/STAT pathway [54]. Within the JAK/STAT pathway, the transcription factors STAT1 and STAT2 become phosphorylated and subsequently form STAT1-STAT1 homodimers and STAT1-STAT2 heterodimers. The dimers translocate into the nucleus to further activate the interferon stimulated genes (ISG) which are responsible for the varied cellular responses. Interferon stimulated genes comprise several hundred genes, which can be grouped into ISRE-dependent genes, induced by the ISG3 complex (formed by STAT1-STAT2 heterodimers and IRF9), or GAS-dependent genes, induced by STAT1-STAT1 homodimers [55].

In this section we analyze the capacity for multiple steady states at different levels of the interferon (IFN) signaling pathway, from the ligand-receptor interactions at the membrane to the transcriptional level. We use our deficiency-oriented and injectivity-oriented approaches to identify network structures that are (i) compatible with current biological knowledge on the receptor-ligand interactions, the downstream JAK/STAT signaling pathway and its control of gene expression, and (ii) allow for bistability, that is, the existence of multiple positive steady states that reflect the outcomes of a cellular decision process. Detailed computations and additional figures are included in S1 Appendix, and Matlab codes are provided in S1 File. The computation time for finding a solution with global scatter search [52] in networks showing bistability was on the order of 20 to 60 seconds on an Intel 2.8 GHz Xeon processor.

### Interferon-receptor complex formation

**Fig 4:**
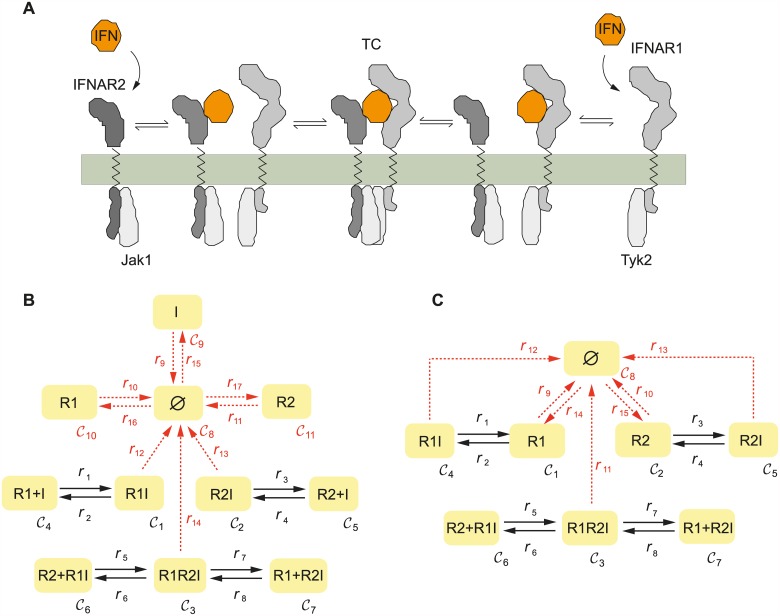
*C*-graph of the ternary complex formation. A) Scheme of the mechanism; B) *C*-graph of the network with 6 species involved in the dynamics; C) *C*-graph of the network when IFN is in excess. Solid arrows correspond to true reactions and dashed arrows represent inflow/outflow reactions.

The formation of the ternary interferon-receptor complex is among the best studied components of the interferon pathway [38]. The model we consider comprises the receptor-ligand interactions occurring on the cell membrane and, more precisely, the binding of extracellular interferon to one of the interferon receptor subunits (IFNAR1 or IFNAR2) and the subsequent recruitment of the other receptor subunit to compose the IFN-IFNAR1-IFNAR2 ternary complex (see Fig 4A). The stability of the ternary complex has been experimentally shown to trigger differential biological responses [39], and we therefore investigate whether bistability could be present already at this initial stage of interferon signalling.

#### Ternary complex formation network

First, we consider the mechanism of ternary complex formation as depicted in Fig 4B. considering only the reactions indicated by solid arrows, i.e. those with no inflow/degradation of any of the involved species. The network is reversible (and therefore uniterminal) with three mass conservation laws corresponding to interferon (here denoted by I), the receptor subunit IFNAR1 (R1) and the receptor subunit IFNAR2 (R2) in all their forms. Therefore, we can apply the deficiency-oriented approach to find parameter vectors compatible with bistable behavior (the deficiency of the network is *δ* = 1). No parameter vector was found leading to multistability. This result is compatible with the Deficiency One Algorithm [22], which precludes multistationarity for this network.

#### Ternary complex formation network with IFN in excess

When interferon is in excess over the rest of the species, its concentration can be assumed to be constant, and be embedded into the corresponding kinetic rate. Under this assumption, the resultant network, depicted in Fig 4C (only the reactions indicated by solid arrows) is again of deficiency *δ* = 1. Applying the deficiency-oriented approach we do not find any parameter vector for which the network is bistable. The result is again compatible with the outcome of the Deficiency One Algorithm [22] which precludes multiple steady states for any value of the parameters.

#### Semi-diffusive network with constant IFN inflow

Next, we consider the mechanism of ternary complex formation taking into account the degradation of all the involved species, the basal expression of the receptor subunits IFNAR1 and IFNAR2, and a constant inflow of interferon. The resultant network is of deficiency *δ* = 2. This network has no conservation laws and fulfills the requirements to apply the injectivity-oriented approach. No parameters were found leading to multistationary behavior.

#### Semi-diffusive network with an excess of IFN

Finally, basal expression of the receptor subunits IFNAR1 and IFNAR2 was considered together with an excess of interferon, and the degradation of all the species involved. The resultant network is of deficiency *δ* = 1. Using the same methodology as in the preceding case, no parameters where found leading to multistable behavior.

In summary, none of the topological versions considered for the ternary complex formation was found to show bistable behavior. A test with CoNtRol by Donnell et al. [26] confirms that none of the networks considered in the interferon-receptor complex formation is multistationary. Here it is important to remark that we can not preclude the existence of multiple steady states based on the results obtained by the optimization method. For precluding multistationarity, other injectivity-based methods [26–28, 56] provide conclusive results.

### Early STAT signaling upon interferon stimulation

**Fig 5:**
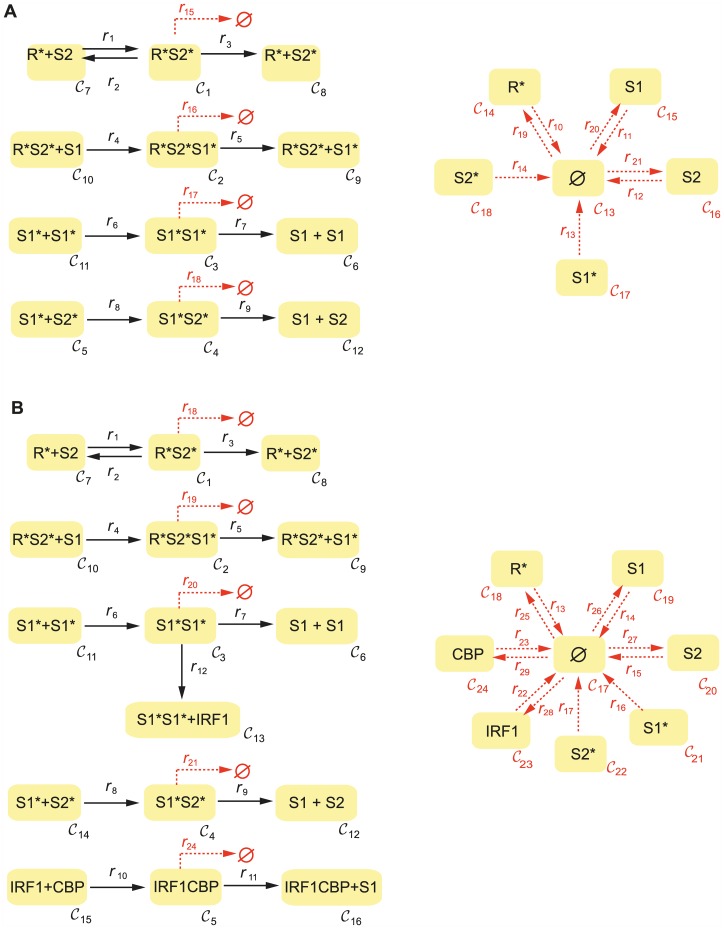
*C*-graphs of the considered models of STAT signaling upon IFN stimulation: A) early STAT signaling, B) STAT signaling including expression of STAT1 via interferon regulatory factor IRF1 and CREB-Binding Protein (CBP). *S*1 = STAT1, *S*2 = STAT2, R*=activated receptor, *S*1*=phosphorylated STAT1, *S*2*=phosphorylated STAT2.

To model signalling processes downstream of the activated receptor-ligand complex, we consider the mechanism depicted in Fig 5A. Taken with mass action kinetics, this mechanism is compatible with the experimental data obtained for the first two hours upon IFN stimulation (included in S1 Appendix) and with current biological knowledge reporting a pivotal role of STAT2 in type I IFN signaling [57].

The JAK/STAT pathway transduces a multitude of signals from various receptors to trigger appropriate gene transcription [58]. Thus, as a shared module, we decided to investigate whether its topological structure could host bistable dynamics.

The model incorporates STAT2 activation through binding to the ternary interferon-receptor complex and a subsequent recruitment and activation of STAT1 [59]. Phosphorylated STATs can further dissociate from the activating complex and form homodimers (STAT1-STAT1) or heterodimers (STAT1-STAT2) that act as transcription factors and modulate biological responses.

#### Closed network

Firstly, we consider the mechanism with no inflow/degradation of any of the species involved. The network is closed (there is a strictly positive row vector in the left kernel of the stoichiometric matrix) and uniterminal. The deficiency is *δ* = 2. We apply the deficiency-oriented approach and find a saddle-node bifurcation, concluding that the system can exhibit multistationarity. First, we find a zero of the objective function *F*_*def*_, where we start a continuation of equilibria obtaining two different equilibrium branches.

#### Semi-diffusive network

Next, we consider the STAT signalling mechanism taking into account the degradation of all species involved and the basal expression of STAT1 and STAT2, as well as a constant input of active receptor (see Fig 5A). The resultant network is of deficiency *δ* = 8. The saddle-node conditions based on the injectivity criterion were checked by global optimization methods [52]. Again we find a set of fluxes making the objective function *F*_*inj*_ zero (the system admits a saddle-node). Starting a continuation from the saddle-node we conclude that bistability is preserved in the open system.

All bifurcation diagrams are included in S1 Appendix.

### STAT signaling and feedback via STAT1 expression

The existence of bistability in a pathway submodule does not necessarily imply that a bigger network containing this submodule is bistable [19]. Next we aim to elucidate whether the bistability is preserved when the STAT pathway is embedded in a broader network including transcriptional feedback. Multiple feedbacks have been uncovered in JAK/STAT signalling, including inhibition by suppressor of cytokine signaling (SOCS) [60], USP18 negative feedback control [61] or STAT1 expression. We consider here the expression of STAT1 via interferon regulatory factor IRF1 and CREB-binding protein (CBP), as postulated by Smieja et al. [55]. We examine the mechanism depicted in Fig 5B, considering basal expression of STAT1, STAT2, IRF1, CBP and a constant inflow of activated receptor, as well as the degradation of all species.

We found a set of fluxes fulfilling the sufficient conditions for a saddle-node based on the injectivity criterion. Starting a continuation from this point we find that in fact, the STAT network extended to include the STAT1 feedback, preserves its bistability. Bistability of the network in Fig 5B also follows from Theorem 5.1 in [62].

### Receptor complex formation and STAT signaling

**Fig 6:**
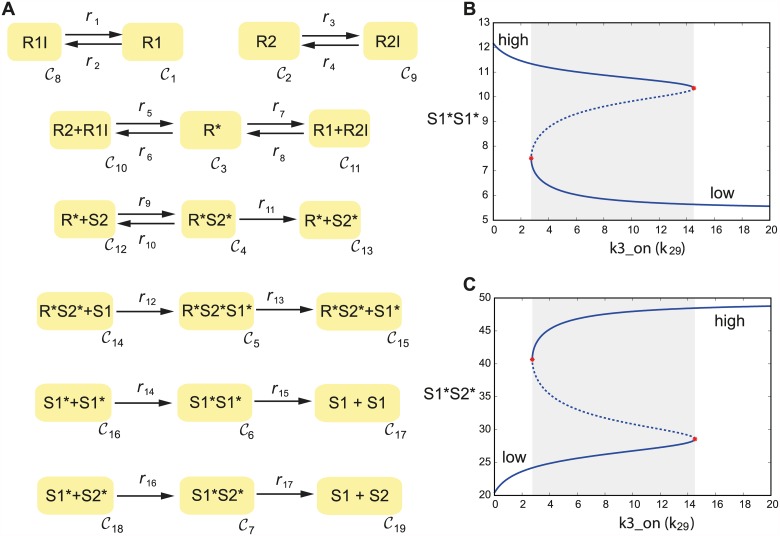
A) *C*-graph of the early STAT signaling network coupled with the receptor complex formation upon IFN induction and corresponding bifurcation diagrams (B-C) where the steady state concentration of the transcription factor is depicted against the interferon association rate constant *k*_29_.

Finally, we analyze the early STAT signaling network coupled with the receptor complex formation network upon IFN induction. The complete module is depicted in Fig 6A. Using the deficiency-oriented approach the network is found to be bistable. This example shows how our method is complementary to existing theory: bistability of the network also follows from Theorem 4.2 in [29], and our method provides parameter sets leading to bistable behaviour. We take one set of parameters found by our algorithm which fulfills the saddle-node condition, and perform a bifurcation analysis using standard tools [53]. We observe that, by varying a parameter related to the affinity of the receptor towards interferon (*k*_29_), we obtain a discontinuous jump in the steady state levels of the proteins involved, showing hysteretic behavior and a rather broad range of bistability. Below a threshold in the parameter value, the system is in a steady state with high level of the STAT1-STAT1 transcription factor and low level of STAT1-STAT2, while above the threshold the situation is reversed (low STAT1-STAT1, high STAT1-STAT2). We observe also a threshold behavior with respect to the initial levels of STATs and IFN receptors (bifurcation diagrams are included in S1 Appendix).

## Discussion

Reaction networks, ranging from chemical species interactions in stirred tank reactors to molecular pathways within cells, can be classified according to the exchange of matter with the *environment* through the (real or imaginary) boundaries of the volume where the reactions are taking place. In cell signaling, these fluxes of matter can be of the form of protein basal formation, degradation or translocation from/to a different compartment. One fundamental difference with respect to the Continuous Flow Stirred Tank Reactor (CFSTR) paradigm is the fact that, due to the presence of protein complexes, not all the species can be in the inlet/input flow. To overcome this, we define the class of semi-diffusive networks to which the majority of signaling pathways without mass conservation can be easily adapted.

In order to provide adequate methods for detecting sources of bistability in signaling, we take into account the particularities of signaling models (both with and without mass conservation). We formulate the search for multistationarity as an algorithm, in which first global optimization methods efficiently search for saddle-node points in a signaling pathway of interest, identifying decision variables that cause a saddle-node (and potentially multiple steady states) to occur. These decision variables contain the kinetic rate constants for systems with mass conservation, and the reaction fluxes for systems without mass conservation (in this case a compatible set of kinetic constants and protein steady state values is retrieved from the reaction fluxes). If a saddle-node is found, in a second step we start a continuation of equilibrium in forward and backward directions using standard bifurcation tools [53] such that, provided that the saddle-node is a saddle-node bifurcation, two branches of equilibria are automatically computed. One interesting aspect of the approaches presented is that we can incorporate all the quantitative information available regarding experimental conditions, kinetic constants, etc. by fixing known kinetic parameters, protein concentrations or reaction fluxes at the steady state. This is particularly useful in the context of integrated experimental-computational modeling approaches when we are interested in detecting sources of bistability under specific biological/experimental conditions.

Global optimization solvers provide us both with computational scalability to handle high dimensional state and parameter spaces typical in the context of modeling signaling pathways, and computational speed to find solutions in the order of seconds. As a drawback, if the algorithm does not find a zero of the objective function we cannot formally preclude the existence of saddle-nodes and therefore multiple steady states. Non-heuristic search strategies like interval methods will provide conclusive results [37] but they become computationally untractable in realistic signaling scenarios.

Our approach was motivated by the need of evaluating bistability sources at different levels during the modeling process of the interferon signaling pathway. Although Chemical Reaction Network Theory appeared to be a powerful framework, to the best of our knowledge, there was no unified method for multi-stationarity detection based on CRNT that provides parameter sets leading to multiple steady states and that could also be applied to the majority of signaling pathways. As mentioned in the introduction, other methods for detection of complex nonlinear behavior in biochemical reaction networks based on different network graphs have been developed recently, including the software GraTelPy by Walther et al. [25] which uses graph-theoretic analysis of the bipartite graph to find sources of multistability, oscillations or Turing instabilities, and the toolbox CoNtRol by Donnell et al. based on the DSR graph [26]. Importantly, the two methods presented here can be fully automated in an algorithmic manner, combining numerical with simple symbolic computations (basically simple symbolic derivatives). Moreover, the formulation of the saddle node detection as an optimization problem allows to exploit the efficiency of global optimization solvers. In terms of network size, this ensures good scalability to large networks. The implementation of a Matlab toolbox is subject of ongoing work. Our approach has broad applicability, since, as stated in the Methods section, the assumptions that we need (namely mass action kinetics, the existence of a positive steady state, a uniterminal graph in case of networks with mass conservation, and semi-diffusive regime in case of no mass conservation) are mild in the context of cell signaling.

Regarding the biology of type I interferons, the following open questions have attracted the attention of the community: why are there many different interferon subtypes (for example, 16 subtypes in the IFN I family in humans) and how can different signaling outcomes be generated through the same receptor. Depending on the type/dose of interferon and the cell context, the cell outcome might vary from antiviral to apoptotic activity. It has been shown that differences in affinity to the receptor subunits, through a different stability of the ternary complex, dictate differential biological activities [39] but the underlying signaling mechanisms are not understood.

Our analysis of network topologies shows that bistability appears already at the level of early STAT signaling, and that varying parameters related to the interferon input and affinity, the system can decide (in a threshold fashion) between two different outcomes, characterized by different levels of active transcription factors coding for two families of genes (ISRE and GAS). We also observe threshold behavior with respect to the initial levels of proteins within the cell. In this way, the STAT signaling pathway could be translating input/cell context differentials into different response patterns, contributing to explain the observed differential signaling. The analysis of the interferon pathway demonstrates that CRNT-based methods can help understanding realistic signaling pathway representations; it opens a promising line for further investigation concerning both STAT and IFN signaling by combining *in-silico* and *in-vitro* approaches.

## Supporting information

S1 Appendix Supporting proofs and computations. Pdf file containing supporting definitions, proofs, detailed computations and additional figures.
(PDF)

S1 File Matlab codes. Zip file containing Matlab codes.
(ZIP)
