# Supplementary material for: Chemical Reaction Network Theory elucidates sources of multistability in interferon signaling
Source: PLoS Comput Biol. 2017 Apr 3;13(4):e1005454. doi: 10.1371/journal.pcbi.1005454 (PMC5400276; doi:10.1371/journal.pcbi.1005454)
Supplement: S1 Appendix — Pdf file containing supporting definitions, proofs, detailed computations and additional figures. (PDF) [file pcbi.1005454.s001.pdf]

Chemical Reaction Network Theory Elucidates Sources of Multistability in Interferon Signaling. S1 Appendix

I. Otero-Muras, P. S. Yordanov, and J. Stelling

Contents

1 Some definitions and results from bifurcation analysis **2**
1.1 Saddle-node and saddle-node bifurcation 2
1.2 Saddle-node bifurcation, multistationarity and multistability . 4

2 Sufficient condition for a saddle-node in reaction networks with mass conservation **6**

3 Sufficient condition for a saddle-node in semi-diffusive reaction networks **11**

4 Interferon-receptor complex formation **15**
4.1 Closed ternary complex formation network 15
4.2 Ternary complex formation network with IFN in excess 17
4.3 Semi-diffusive network with constant IFN inflow 19
4.4 Semi-diffusive network with IFN in excess 21

5 Early STAT signaling upon interferon stimulation **23**
5.1 Closed network 23
5.2 Semi-diffusive network 27

6 STAT signaling and feedback via STAT1 expression **30**

7 Receptor complex formation and STAT signaling **34**

8 STAT activation dynamics upon IFN stimulation **41**

Chapter 1

Some definitions and results from bifurcation analysis

1.1 Saddle-node and saddle-node bifurcation

We follow [1] to define the concepts of ***limit point*** (or ***saddle-node***) and ***limit point bifurcation*** (also known as ***saddle-node bifurcation***, ***tangent bifurcation***, ***fold bifurcation*** or ***turning point***) in the context of bifurcation analysis, and explain their implications for the existence of multiple steady states in nonlinear ODE systems.

Consider the system of ODEs:

$$\dot{u} = f(u, \beta) \qquad u \in \mathbb{R}^n, \beta \in \mathbb{R} \tag{1.1}$$

where u is the state vector, β is a real parameter, and the function $f : \mathbb{R}^n \times \mathbb{R} \to \mathbb{R}^n$ is smooth.

Let us define

$$x = \begin{pmatrix} u \\ \beta \end{pmatrix} \in \mathbb{R}^{n+1},$$

we say that the system (1.1) is at equilibrium if:

$$F(x) \equiv f(u, \beta) = 0. \tag{1.2}$$

The set of points $x = (u^T, \beta)^T$ satisfying Eq. (1.2) constitutes the equilibrium manifold in the (u, β)-space. An ***equilibrium continuation*** consists of computing a solution set $M \subset \mathbb{R}^{n+1}$ of the smooth system (1.2) starting from a given point $x_0 \in M$.

Let Q be the $n \times (n+1)$ matrix:

$$Q(u,\beta) = [D_u f \;\; D_\beta f] = \begin{pmatrix} \frac{\partial f_1}{\partial u_1} & \frac{\partial f_1}{\partial u_2} & \cdots & \frac{\partial f_1}{\partial u_n} & \frac{\partial f_1}{\partial \beta} \\ \frac{\partial f_2}{\partial u_1} & \frac{\partial f_2}{\partial u_2} & \cdots & \frac{\partial f_2}{\partial u_n} & \frac{\partial f_2}{\partial \beta} \\ & & \cdots & & \\ \frac{\partial f_n}{\partial u_1} & \frac{\partial f_n}{\partial u_2} & \cdots & \frac{\partial f_n}{\partial u_n} & \frac{\partial f_n}{\partial \beta} \end{pmatrix}. \quad (1.3)$$

Next we introduce the concept of ***regular point***.

Definition 1 [1] A point $(u^{*T}, \beta^*)^T$ satisfying (1.2), i.e, $f(u^*, \beta^*) = 0$, is called ***regular*** if $\text{rank}(Q(u^*, \beta^*)) = n$.

As a consequence of the *Implicit Function Theorem* the following result holds:

Lemma 1 [1] Near any regular point $(u^{*T}, \beta^*)^T$, Eq. (1.2) defines a solution curve M that passes through $(u^{*T}, \beta^*)^T$ and is locally unique and smooth.

The proof of Lemma 1 can be found in [1].

The following result introduces some important properties of the tangent vector to the solution curve at a regular point:

Lemma 2 [1] A tangent vector v to the solution curve M at a regular point $(u^{*T}, \beta^*)^T \in M$ satisfies $Qv = 0$. If $(u^{*T}, \beta^*)^T$ is a regular point for (1.2) then the linear equation $Qv = 0$ with $Q = [D_u f \;\; D_\beta f]$ has a unique (modulo scaling) solution $v \in \mathbb{R}^{n+1}$, i.e. the kernel of Q is one-dimensional.

The proof of Lemma 2 can be found in [1].

Next, we introduce the concept of ***limit point*** (or ***saddle-node***).

Definition 2 [1] A regular point $(u^{*T}, \beta^*)^T$ is a ***limit point*** for (1.2) with respect to β if $v_{n+1} = 0$, where v is a normalized tangent vector to M at $(u^{*T}, \beta^*)^T$.

Definition 3 [3] A point $(u^{*T}, \beta^*)^T$ is a ***limit point bifurcation*** (also known as ***saddle-node bifurcation***, ***fold bifurcation***, or ***turning point***) of (1.2) with respect to β if the following conditions hold:

1. $(u^{*T}, \beta^*)^T$ is a limit point of (1.2) with respect to β,
2. there exists a parametrization $u(s)$, $\beta(s)$ with $u(s_0) = u^*$, $\beta(s_0) = \beta^*$, and $d^2\beta(s_0)/ds^2 \neq 0$.

Condition (2) prevents $(u^{*T}, \beta^*)^T$ from being a *hysteresis point* [3] and can be replaced by other equivalent *non degeneracy* conditions, including conditions

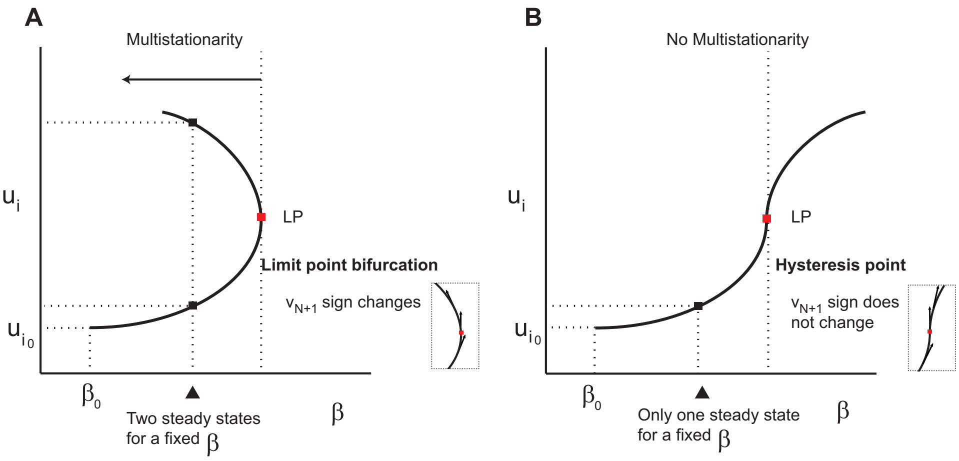

Figure 1.1: Bifurcation diagrams corresponding to A) a limit point bifurcation and B) a hysteresis point (or *degenerate* limit point).

2b and 2c introduced next. Let us assume that $(u^{*T}, \beta^*)^T$ is a limit point of (1.2) with respect to β (i.e. condition 1 is fulfilled):

Condition 2b (From [4], Saddle-Node Bifurcation Theorem). Suppose that the kernel of the linear transformation $D_u f(u^*, \beta^*) : \mathbb{R}^n \to \mathbb{R}^n$ is spanned by the nonzero vector $w \in \mathbb{R}^n$. Let $D^2_{uu} f(u^*, \beta^*)(w, w)$ be the second Fréchet derivative[1] of f evaluated at $(u^{*T}, \beta^*)^T$ in the directions given by w and w. If $D^2_{uu} f(u^*, \beta^*)(w, w) \in \mathbb{R}^n$ is non zero and not in the range of $D_u f(u^*, \beta^*)$, the limit point $(u^{*T}, \beta^*)^T$ is a limit point bifurcation.

Condition 2c Let v be the tangent vector to the equilibrium curve at $(u^T, \beta)^T$, if during an equilibrium continuation, the sign of v_{n+1} (there exists a parametrization of M that makes v_{n+1} zero) changes as we pass through $(u^{*T}, \beta^*)^T$, the limit point $(u^{*T}, \beta^*)^T$ is also a limit point bifurcation [5].

Fig. 1.1 illustrates the concepts of limit point bifurcation and hysteresis point.

1.2 Saddle-node bifurcation, multistationarity and multistability

If the point $(u^{*T}, \beta^*)^T$ is a limit point bifurcation for (1.2) with respect to the parameter β the dynamic system (1.1) has ***multiple steady states***, see Fig. 1.1.

[1]Fréchet derivative generalizes the notion of gradient to multivariate matrix functions.

If the point $(u^{*T}, \beta^*)^T$ with u^* being strictly positive is a limit point bifurcation for (1.2) with respect to the parameter β, the dynamic system (1.1) has ***multiple positive steady states***, i.e. the system is ***multistationary***.

Here it is important to note that situations in which a limit point is not a limit point bifurcation (turning point) are quite exceptional in practice.

Multiple steady states (although not frequently) might not imply ***multistability*** (multiple stable steady states).

Starting a continuation of equilibrium from a limit point (or saddle-node) in forward and backward directions [5] we elucidate whether the limit point is a saddle-node bifurcation or a hysteresis point. If the point is a saddle-node bifurcation, two branches of equilibria emerge, one stable and one unstable. In order to check whether a multistationary system is multistable, we continue the equilibrium curve until we find a second saddle-node bifurcation.

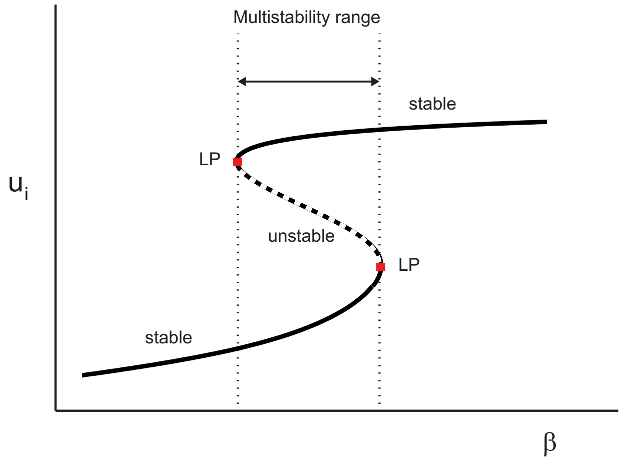

Figure 1.2: Bifurcation diagram of a multistable system.

Chapter 2

Sufficient condition for a saddle-node in reaction networks with mass conservation

For a reaction network with N species in M complexes, participating in R reactions endowed with mass action kinetics, the dynamics are given by a system of N ODEs of the form:

$$\dot{c} = YA\psi(c) = Sv(c,k) \tag{2.1}$$

where $c \in \mathbb{R}^N$ is the vector of concentrations of the species involved, A is a $M \times M$ matrix containing kinetic constants constructed as indicated in the main text and $\psi(c) \in \mathbb{R}^M$ is the vector of mass action monomials. Matrices Y and S are the molecularity and stoichiometric matrices respectively, also introduced in the main text.

Let us denote by ℓ the number of linkage classes of the graph of complexes, and by $\mathcal{D}$ the deficiency subspace (of dimension δ) of the network.

In this section we consider reaction networks in presence of mass conservation fulfilling assumptions in the main text, i.e.:

A.1 Mass action kinetics.

A.2 Capacity for a positive equilibria.

A.3 Uniterminal graph of complexes.

The locus of equilibria of (2.1) can be expressed by a set of $M - \ell$ algebraic equations of the form:

$$\mathcal{H}(c, \alpha, k) = 0, \tag{2.2}$$

where $\alpha \in \mathbb{R}^{\delta}$ and $k \in \mathbb{R}^{R}$ are the deficiency and kinetic parameter vectors, respectively. See the main text for more details on how $\mathcal{H}(c, \alpha, k)$ is computed. Detailed computations are included for all the examples in following chapters of this S1 Appendix (4.1, 4.2, 5.1 and 7).

Let s be the rank of the stoichiometric matrix. For networks with conservation relationships ($N - s > 0$) in which each conservation relation represents conservation of a chemical unit or moiety the (mass) conservation relationships are given by a set of $\lambda = N - s$ algebraic equations of the form:

$$W(c, \sigma) = 0, \tag{2.3}$$

where $W(c, \sigma) = B^T c - \sigma$, $\sigma = B^T c_0$ and c_0 is a reference concentration vector. For a given σ the reaction polyhedron is defined as the set of $c \geq 0$ fulfilling Eq. (2.3).

Each mass conservation relationship in (2.3) describes the conservation of a particular *moiety*. The set of conserved moieties of the reaction network is denoted by $\mathscr{M}$:

$$\mathscr{M} = \{\mathcal{M}_1, \ldots, \mathcal{M}_{\lambda}\}.$$

The set of equations

$$\begin{aligned} \mathcal{H}(c, \alpha, k) &= 0 \\ W(c, \sigma) &= 0 \end{aligned} \tag{2.4}$$

defines the locus of equilibria of the system (2.1) compatible with the reaction polyhedron fixed by $\sigma \in \mathbb{R}^{\lambda}_{>0}$. Note that (2.4) contains $N + \delta$ equations [6].

Let us compute the matrix:

$$G(c, \alpha, k) = \begin{pmatrix} D_c\mathcal{H} & D_{\alpha}\mathcal{H} \\ D_c W & D_{\alpha} W \end{pmatrix}. \tag{2.5}$$

Matrix G is square of dimensions $N + \delta$. Taking into account that $D_c W = B^T$ and $D_{\alpha} W = 0$, G reads:

$$G(c, \alpha, k) = \begin{pmatrix} D_c\mathcal{H} & D_{\alpha}\mathcal{H} \\ B^T c & 0 \end{pmatrix}. \tag{2.6}$$

When we fix the parameters α and k, we can write (2.4) in the form:

$$f(u, \sigma_i) = 0 \tag{2.7}$$

where σ_i is the mass conservation constant corresponding to the conserved moiety $\mathcal{M}_i$ (the conservation constants corresponding to the remaining moieties in $\mathscr{M}$ are also fixed), u is given by:

$$u = \begin{pmatrix} c \\ \alpha \end{pmatrix} \in \mathbb{R}^{N+\delta}, \tag{2.8}$$

and the function $f : \mathbb{R}^{N+\delta} \times \mathbb{R} \to \mathbb{R}^{N+\delta}$ is smooth.

Proposition 1 Let us consider a reaction network fulfilling assumptions A.1, A.2 and A.3, with dynamics described by (2.1) where moieties $\mathcal{M}_1, \ldots, \mathcal{M}_\lambda$ are being conserved.

If there are $c^* \in \mathbb{R}^N_{>0}$, $\alpha^* \in \mathbb{R}^\delta$ and $k^* \in \mathbb{R}^R_{>0}$ such that

$$\mathcal{H}(c^*, \alpha^*, k^*) = 0, \tag{2.9}$$
$$D_c\mathcal{H}(c^*, \alpha^*, k^*) \text{ is of full rank,} \tag{2.10}$$
$$\text{rank}(G(c^*, \alpha^*, k^*)) = N + \delta - 1 \text{ with } G \text{ defined by (2.5),} \tag{2.11}$$

then, for $k = k^*$ and any $i = 1, \ldots, \lambda$, Eq. (2.7) has a limit point at $(u^{*T}, \sigma_i^*)^T$ with respect to the parameter σ_i (where σ_i is the mass conservation constant of the moiety $\mathcal{M}_i \in \mathscr{M}$).

Proof: Let us take a moiety $\mathcal{M}_i \in \mathscr{M}$, and express Eq. (2.4) in an equivalent form according to Eq. (2.7).

Note that (2.7) is of the form of Eq. (1.2) with u defined in Eq. (2.8), $\beta = \sigma_i$ and $n = N + \delta$.

Starting from (2.7) we can compute the matrix Q (1.3) as:

$$Q = [D_u f \;\; D_{\sigma_i} f] = [G \;\; D_{\sigma_i} f], \tag{2.12}$$

where Q is of dimensions $(N + \delta) \times (N + \delta + 1)$. By (2.11) the rank of G is equal to $N + \delta - 1$. We can partition $D_{\sigma_i} f$ as follows:

$$D_{\sigma_i} f = \begin{bmatrix} D_{\sigma_i}\mathcal{H} \\ D_{\sigma_i} W \end{bmatrix} \tag{2.13}$$

where, as it can be deduced from (2.4), $D_{\sigma_i}\mathcal{H} = 0$ and

$$D_{\sigma_i} W = \begin{pmatrix} \frac{\partial W_1}{\partial \sigma_i} \\ \vdots \\ \frac{\partial W_\lambda}{\partial \sigma_i} \end{pmatrix}$$

with $\partial W_j/\partial \sigma_i$ being -1 for $j = i$ and zero otherwise. Vector $D_{\sigma_i}W$ has thus one and only one entry different from zero.

Note that G can be partitioned as:

$$G = \left[\begin{array}{c|c} D_c\mathcal{H} & D_\alpha\mathcal{H} \\ \hline B^T & 0 \end{array}\right] = \left[\begin{array}{c|c} D_c f & D_\alpha f \end{array}\right]$$

and so the matrix Q reads:

$$Q = \left[\begin{array}{c|c|c} D_c\mathcal{H} & D_\alpha\mathcal{H} & 0 \\ \hline B^T & 0 & D_{\sigma_i}W \end{array}\right] = \left[\begin{array}{c|c|c} D_c f & D_\alpha f & D_{\sigma_i} f \end{array}\right].$$

For any $i = 1, \dots, \lambda$, the vector $D_{\sigma_i}f$ is linearly independent of the columns of $D_\alpha f$.

Taking into account that by (2.10) $D_c\mathcal{H}$ is full rank, the vector $D_{\sigma_i}f$ (for any $i = 1, \dots, \lambda$) is also linearly independent of the columns of $D_c f$, and thus, since $\text{rank}(G) = N + \delta - 1$, we have that $\text{rank}(Q) = N + \delta$.

Therefore, according to Lemma 1, for any $\mathcal{M}_i \in \mathscr{M}$, $f(u, \sigma_i) = 0$ defines a locally unique and smooth curve passing through $(u^{*T}, \sigma_i^*)^T$.

Let $v \in \mathbb{R}^{N+\delta+1}$ be the tangent vector to the curve at $(u^{*T}, \sigma_i^*)^T$ and decompose the product Qv into:

$$Qv = Gw \ + \ gv_{N+\delta+1}, \tag{2.14}$$

where

$$v = \begin{pmatrix} w \\ v_{N+\delta+1} \end{pmatrix}$$

and $g = D_{\sigma_i}f$. Computed in (2.13), $D_{\sigma_i}f$ has a strictly negative entry (corresponding to moiety $\mathcal{M}_i$). Therefore, $gv_{N+\delta+1} = 0$ if and only if $v_{N+\delta+1} = 0$.

On the other hand, according to Lemma 2, $Qv = 0$, with $v \in \mathbb{R}^{N+\delta+1}$ being unique (modulo scaling).

The rank of G is $N + \delta - 1$ by (2.11), and therefore a vector in its kernel is also unique (modulo scaling). Then, $Qv = 0$ implies $Gw = 0$ where $v = (w^T \ \ v_{N+\delta+1})^T$. Consequently, since we also have that $gv_{N+\delta+1} = 0$ if and only if $v_{N+\delta+1} = 0$, we can deduce that $v_{N+\delta+1} = 0$. Therefore, according to Definition 2, $(u^{*T}, \sigma_i^*)^T$ is a limit point for (2.7) with respect to the parameter σ_i (for any $i = 1, \dots, \lambda$).

Remark 1 For convenience, we have proven $(c^{*T}, \sigma_i^*)^T$ to be a limit point for (2.7) with respect the mass conservation constant σ_i, which does not

preclude $(c^{*T}, \beta^*)^T$ from being a limit point for (2.7) with respect to other parameter β chosen from the set of kinetic constants.

Remark 2 With a slight abuse of notation, if $(c^{*T}, \sigma_i^*)^T$ is a limit point for (2.7) (with $k = k^*$) with respect the mass conservation constant σ_i (or with respect to any other parameter), we say that the system (2.4) (and the associated reaction network) has a saddle-node at c^*, k^*.

Chapter 3

Sufficient condition for a saddle-node in semi-diffusive reaction networks

Let us consider a reaction network fulfilling the following assumptions:

A.1 Mass action kinetics.

A.2 Capacity for a positive equilibria.

A.4 Semi-diffusive network.

The dynamics of such reaction system are given by a set of ODEs of the form:

$$\dot{c} = K + S_{to} v_{to}(c, k) \tag{3.1}$$

as described in the main text, where $c \in \mathbb{R}^N$ is the vector of states, $K \in \mathbb{R}^N_{\geq 0}$ is the constant inflow term, S_{to} is the $N \times R_{to}$ stoichiometric matrix of true and outflow reactions and $v_{to} : \mathbb{R}^N_{\geq 0} \times \mathbb{R}^{R_{to}}_{>0} \to \mathbb{R}^{R_{to}}_{\geq 0}$ defines the reaction rates of true and outflow reactions.

Let us denote the set of involved species by

$$\mathscr{S} = \{\mathcal{S}_1, \ldots, \mathcal{S}_N\}.$$

Let us remind here that we refer by *key* species to the species that are present in the inlet. The system (3.1) is at equilibrium if:

$$0 = K + S_{to} v_{to}(c, k). \tag{3.2}$$

The Jacobian of the system is given by:

$$J(c,k) = S_{to} diag(v_{to}) Y_r^T diag(c^{-1}).$$

where Y_r is the matrix of the source complexes of true and outflow reactions. Let $\mu \in \mathbb{R}^{R_{to}}$ be the vector containing the fluxes of the true and outflow reactions. We define:

$$\overline{J}(\mu) = S_{to} diag(\mu) Y_r^T \tag{3.3}$$

as in the main text.

Let us define the polynomial function

$$p(c,k) = -S_{to} v_{to}(c,k) \tag{3.4}$$

and its counterpart in terms of the fluxes as:

$$\overline{p}(\mu) = -S_{to}\mu. \tag{3.5}$$

The relationship between fluxes and concentrations is given by:

$$\mu = v_{to}(c,k) \tag{3.6}$$

where k denotes here the vector of kinetic constants of true and outflow reactions.

In semi-diffusive networks there is an outflow reaction with rate $k_{o_i} c_i$ for each species $\mathcal{S}_i \in \mathscr{S}$, corresponding to the degradation of species $\mathcal{S}_i$.

We can write (3.2) in an equivalent way as:

$$f(c, k_{o_i}) = 0, \tag{3.7}$$

where k_{o_i} is the degradation constant of species $\mathcal{S}_i \in \mathscr{S}$, the remaining parameters are fixed, and $f : \mathbb{R}^N \times \mathbb{R} \to \mathbb{R}^N$ is smooth.

Proposition 2 Let us consider a reaction network fulfilling assumptions $A.1$, $A.2$ and $A.4$ with dynamics described by Eq. (3.1) and involving the set of species $\mathscr{S}$. Let $\mu \in \mathbb{R}^{R_{to}}_{>0}$ represent the fluxes of true and outflow reactions and $\overline{J}(\mu)$ and $\overline{p}(\mu)$ be as defined by (3.3) and (3.5), respectively.

If there is a vector $\mu^* \in \mathbb{R}^{R_{to}}_{>0}$ such that:

$$\text{rank}(\overline{J}(\mu^*)) = N - 1, \tag{3.8}$$

$$\overline{p}_i(\mu^*) > 0 \quad \text{for } i \text{ being a key species}, \tag{3.9}$$

$$\overline{p}_i(\mu^*) = 0 \quad \text{for } i \text{ not being a key species}, \tag{3.10}$$

then, there are strictly positive c^* and k^* satisfying $\mu^* = v_{to}(c^*, k^*)$, and a species $\mathcal{S}_i \in \mathscr{S}$ such that $(c^{*T}, k^*_{o_i})^T$ is a limit point for (3.7) (with $k = k^*$) with respect to the parameter k_{o_i} (degradation constant of species $\mathcal{S}_i \in \mathscr{S}$).

Proof: Taking into account (3.6), for a given $\mu^* \in \mathbb{R}^{R_{to}}_{>0}$ there are $c^* \in \mathbb{R}^N_{>0}$, $k^* \in \mathbb{R}^{R_{to}}_{>0}$ such that $\mu^* = v_{to}(c^*, k^*)$.

Provided that $\mu^* \in \mathbb{R}^{R_{to}}_{>0}$ fulfills (3.8-3.10), we have that:

$$\operatorname{rank}(J(c^*, k^*)) = N - 1, \tag{3.11}$$

$$p_i(c^*, k^*) \geq 0 \quad \text{for } i \text{ being a key species}, \tag{3.12}$$

$$p_i(c^*, k^*) = 0 \quad \text{for } i \text{ not being a key species}. \tag{3.13}$$

Taking $K^* \in \mathbb{R}^N_{\geq 0}$ such that $K^* = p(c^*, k^*)$ we have:

$$K^* + S_{to} v_{to}(c^*, k^*) = 0$$

and therefore c^* and k^* together with K^* define an equilibrium point of (3.1).

Let us take a species $\mathcal{S}_i \in \mathscr{S}$, and express Eq. (3.2) in an equivalent form according to Eq. (3.7).

Note that (3.7) is of the form of Eq. (1.2) with $u = c$, $\beta = k_{o_i}$ and $n = N$.

Matrix Q in (1.3) is then computed as:

$$Q = [D_c f \quad D_{k_{o_i}} f] = [J \quad D_{k_{o_i}} f], \tag{3.14}$$

where the rank of J evaluated at the steady state given by c^* and k^* is $N-1$, according to (3.11).

We have:

$$D_{k_{o_i}} f = \begin{pmatrix} \frac{\partial f_1}{\partial k_{o_i}} \\ \vdots \\ \frac{\partial f_N}{\partial k_{o_i}} \end{pmatrix}, \tag{3.15}$$

where $\partial f_j / \partial k_{o_i}$ is strictly negative (equal to $-x_i$) for $j = i$ and zero otherwise, i.e., the vector $D_{k_{o_i}} f$ evaluated at a strictly positive equilibrium has one and only one entry different from zero (corresponding to species $\mathcal{S}_i$).

Let us choose a species $\mathcal{S}_i \in \mathscr{S}$ such that $\operatorname{rank}(Q) = N$ at $(c^{*T}, {k_{o_i}}^*)^T$. Note that the N vectors $D_{k_{o_i}} f$ for $i = 1, \ldots, N$ evaluated at the strictly positive

equilibrium given by c^* and k^* span $\mathbb{R}^N$ and therefore, for semi-diffusive networks we can always choose a species $\mathcal{S}_i \in \mathscr{S}$ such that for $\beta^* = k_{oi}^*$, the rank of $Q = [D_c f \;\; D_{k_{o_i}} f]$ evaluated at $c = c^*$ and $k = k^*$ is equal to N.

According to Lemma 1, for $\mathcal{S}_i$ such that $\text{rank}(Q) = N$, $f(c, k_{o_i}) = 0$ defines a locally unique and smooth curve passing through $(c^{*T}, k_{o_i}^*)^T$.

Let $v \in \mathbb{R}^{N+1}$ be the tangent vector to the curve at $(c^{*T}, k_{o_i}^*)^T$ and decompose the product Qv into:

$$Qv = Jw \;+\; gv_{N+1}, \tag{3.16}$$

where

$$v = \begin{pmatrix} w \\ v_{N+1} \end{pmatrix}$$

and $g = D_{k_{o_i}} f$. The vector g, computed in Eq. (3.15), has one and only one entry which is different from zero (the strictly negative entry corresponding to species $\mathcal{S}_i$). Therefore, $gv_{N+1} = 0$ if and only if $v_{N+1} = 0$.

On the other hand, according to Lemma 2, $Qv = 0$, with $v \in \mathbb{R}^{N+1}$ being unique (modulo scaling).

The rank of J is $N - 1$ by (3.11), and therefore a vector in its kernel is also unique (modulo scaling). Then, $Qv = 0$ implies $Jw = 0$ where $v = (w^T \;\; v_{N+1})^T$.

Consequently, and since we also know that $gv_{N+1} = 0$ if and only if $v_{N+1} = 0$, we can deduce that $v_{N+1} = 0$. Therefore, according to Definition 2, $(c^{*T}, k_{o_i}^*)^T$ is a limit point for (3.7) with respect to the parameter k_{o_i}.

Remark 3 For convenience, we have proven $(c^{*T}, k_{o_i}^*)^T$ to be a limit point for (3.7) with respect to the degradation constant k_{o_i}, which does not preclude $(c^{*T}, \beta^*)^T$ from being a limit point for (3.7) with respect to any other parameter β chosen from the set of kinetic constants (including inflow, true and the remaining outflow reactions).

Remark 4 With a slight abuse of notation, if $(c^{*T}, k_{o_i}^*)^T$ is a limit point for (3.7) (with $k = k^*$) with respect the degradation constant k_{o_i}(or with respect to any other parameter), we say that the system (3.7) (and the associated reaction network) has a saddle-node at c^*, k^*.

Chapter 4

Interferon-receptor complex formation

4.1 Closed ternary complex formation network

There are $N = 6$ species participating in the network with concentrations $c_1 = [I]$, $c_2 = [R1]$, $c_3 = [R2]$, $c_4 = [R1I]$, $c_5 = [R2I]$ and $c_6 = [R1R2I]$. The complexes are labeled such that $\mathcal{C}_1 = R1I$, $\mathcal{C}_2 = R2I$, $\mathcal{C}_3 = R1R2I$, $\mathcal{C}_4 = R1 + I$, $\mathcal{C}_5 = R2 + I$, $\mathcal{C}_6 = R2 + R1I$ and $\mathcal{C}_7 = R1 + R2I$ ($M = 7$).

The molecularity matrix is:

$$Y = \begin{pmatrix} 0 & 0 & 0 & 1 & 1 & 0 & 0 \\ 0 & 0 & 0 & 1 & 0 & 0 & 1 \\ 0 & 0 & 0 & 0 & 1 & 1 & 0 \\ 1 & 0 & 0 & 0 & 0 & 1 & 0 \\ 0 & 1 & 0 & 0 & 0 & 0 & 1 \\ 0 & 0 & 1 & 0 & 0 & 0 & 0 \end{pmatrix}, \tag{4.1}$$

and taking mass action kinetics, the vector of mass action monomials reads:

$$\psi(c) = \begin{pmatrix} c_4, & c_5, & c_6, & c_1c_2, & c_1c_3, & c_3c_4, & c_2c_5 \end{pmatrix}^T.$$

The stoichiometric matrix is:

$$S = \begin{pmatrix} 1 & -1 & 1 & -1 & 0 & 0 & 0 & 0 \\ 1 & -1 & 0 & 0 & 0 & 0 & 1 & -1 \\ 0 & 0 & 1 & -1 & 1 & -1 & 0 & 0 \\ -1 & 1 & 0 & 0 & 1 & -1 & 0 & 0 \\ 0 & 0 & -1 & 1 & 0 & 0 & 1 & -1 \\ 0 & 0 & 0 & 0 & -1 & 1 & -1 & 1 \end{pmatrix},$$

and has rank(S)=3. Therefore, the dimension of the stoichiometric subspace is $s = 3$, and the formula for the deficiency of the network gives:

$$\delta = 7 - 3 - 3 = 1.$$

On the other hand, the dimension of the equilibrium manifold is:

$$\lambda = 6 - 3 = 3.$$

The network is, therefore, under-dimensioned ($\lambda > \delta$). There are three mass conservation laws, defined by $W(c, \sigma) = 0$, where $W(c, \sigma) = B^T c - \sigma$, $\sigma = B^T c$, and

$$B^T = \begin{pmatrix} 1 & 0 & 0 & 1 & 1 & 1 \\ 0 & 1 & 0 & 1 & 0 & 1 \\ 0 & 0 & 1 & 0 & 1 & 1 \end{pmatrix}.$$

Note that the strictly positive vector $\vartheta = (1, 1, 1, 2, 2, 3)$ fulfills $\vartheta S = 0$, as it corresponds to a closed network. We build the matrix Λ in which the entry (i, j) corresponds to the node $\mathcal{C}_i$ in the linkage class $\mathcal{L}_j$, such that:

$$\Lambda_{i,j} = \begin{cases} 1 & \text{if } \mathcal{C}_i \in \mathcal{L}_j \\ 0 & \text{otherwise.} \end{cases}$$

and we get:

$$\Lambda^T = \begin{pmatrix} 1 & 0 & 0 & 1 & 0 & 0 & 0 \\ 0 & 1 & 0 & 0 & 1 & 0 & 0 \\ 0 & 0 & 1 & 0 & 0 & 1 & 1 \end{pmatrix}.$$

Starting from the stoichiometric matrix Y and the matrix Λ we compute a basis for the deficiency subspace using Eq. (8) in the main text, obtaining:

$$\omega = \begin{pmatrix} 1 & -1 & 0 & -1 & 1 & -1 & 1 \end{pmatrix}^T.$$

Therefore we can write $A\psi$ as in Eq. (9) in the main text, obtaining a set of $M - \ell$ linearly independent equations of the form:

$$\begin{aligned} -k_{14}\psi_1 + k_{41}\psi_4 &= \alpha_1 \\ -k_{25}\psi_2 + k_{52}\psi_5 &= -\alpha_1 \\ k_{36}\psi_3 - k_{63}\psi_6 &= -\alpha_1 \\ k_{37}\psi_3 - k_{73}\psi_7 &= \alpha_1. \end{aligned}$$

After substituting the elements of vector ψ by the corresponding monomials we obtain the following expression for the locus of equilibria:

$$\begin{aligned} c_1c_2 - (k_{14}/k_{41})c_4 - \alpha_1/k_{41} &= 0 \\ c_1c_3 - (k_{25}/k_{52})c_5 + \alpha_1/k_{52} &= 0 \\ c_3c_4 - (k_{36}/k_{63})c_6 - \alpha_1/k_{63} &= 0 \\ c_2c_5 - (k_{37}/k_{73})c_6 + \alpha_1/k_{73} &= 0. \end{aligned}$$

Starting from these equations we obtain:

$$c_4 = c_1c_2/(k_{14}/k_{41}) + \alpha_1/k_{41}$$
$$c_3 = -\left(c_2\alpha_1/k_{25} + \alpha_1k_{37}/(k_{73}k_{36}) + \alpha_1/k_{73}\right) / \left(c_1c_2k_{52}/k_{25} - c_4k_{63}k_{37}/(k_{73}k_{36})\right)$$
$$c_6 = c_3c_4/(k_{36}/k_{63}) - \alpha_1/k_{36}$$
$$c_5 = c_1c_3/(k_{25}/k_{52}) + \alpha_1/k_{25}.$$

From Eq. (13) in the main text we get the following matrix G:

$$G = \begin{pmatrix} c_2 & c_1 & 0 & 0 & -k_{14}/k_{41} & 0 & -1/k_{41} \\ c_3 & 0 & c_1 & -k_{25}/k_{52} & 0 & 0 & 1/k_{52} \\ 0 & 0 & c_4 & 0 & c_3 & -k_{36}/k_{63} & -1/k_{63} \\ 0 & c_5 & 0 & c_2 & 0 & -k_{37}/k_{73} & 1/k_{73} \\ 1 & 0 & 0 & 1 & 1 & 1 & 0 \\ 0 & 1 & 0 & 0 & 1 & 0 & 0 \\ 0 & 0 & 1 & 1 & 0 & 1 & 0 \end{pmatrix},$$

of dimensions 7×7, $(N + \delta = 7)$. We formulate an optimization problem to find parameter vectors causing the determinant of G to vanish. The objective function is $F_{def} = det(G)^2$ and we use the following decision vector:

$$\mathrm{x} = (k_{41},\ k_{14},\ k_{25},\ k_{52},\ k_{63},\ k_{36},\ k_{37},\ k_{73},\ \alpha_1, c_1, c_2).$$

We solve the optimization problem (Eq. 14 in the main text) subject to positive concentration vectors (and the equilibrium manifold equations) using the global scatter search algorithm by Egea et al. [7]. We use the implementation of the enhanced scatter search solver (eSS) in the MEIGO toolbox [8], which is freely available at `http://www.iim.csic.es/ gingproc/meigo.html`.

No parameter vector was found for which the objective function becomes zero. The Matlab code is provided in S1 file (`Matlab_code/IFN_receptor/Variant1`).

4.2 Ternary complex formation network with IFN in excess

There are $N = 5$ species participating in the network with concentrations $c_1 = [R1]$, $c_2 = [R2]$, $c_3 = [R1R2I]$, $c_4 = [R1I]$ and $c_5 = [R2I]$. The complexes are labeled such that $\mathcal{C}_1 = R1$, $\mathcal{C}_2 = R2$, $\mathcal{C}_3 = R1R2I$, $\mathcal{C}_4 = R1I$, $\mathcal{C}_5 = R2I$, $\mathcal{C}_6 = R2 + R1I$ and $\mathcal{C}_7 = R1 + R2I$, $(M = 7)$. The molecularity

matrix is:

$$Y = \begin{pmatrix} 1 & 0 & 0 & 0 & 0 & 0 & 1 \\ 0 & 1 & 0 & 0 & 0 & 1 & 0 \\ 0 & 0 & 1 & 0 & 0 & 0 & 0 \\ 0 & 0 & 0 & 1 & 0 & 1 & 0 \\ 0 & 0 & 0 & 0 & 1 & 0 & 1 \end{pmatrix}, \tag{4.2}$$

and the vector of mass action monomials:

$$\psi(c) = \begin{pmatrix} c_1, & c_2, & c_3, & c_4, & c_5, & c_2c_4, & c_1c_5 \end{pmatrix}^T.$$

The stoichiometric matrix is:

$$S = \begin{pmatrix} -1 & 1 & 0 & 0 & 0 & 0 & 1 & -1 \\ 0 & 0 & -1 & 1 & 1 & -1 & 0 & 0 \\ 0 & 0 & 0 & 0 & -1 & 1 & -1 & 1 \\ 1 & -1 & 0 & 0 & 1 & -1 & 0 & 0 \\ 0 & 0 & 1 & -1 & 0 & 0 & 1 & -1 \end{pmatrix}.$$

The rank of the matrix S (and, therefore, the dimension of the stoichiometric subspace) is $s = 3$. We compute the deficiency according to the formula given in the main text as:

$$\delta = 7 - 3 - 3 = 1.$$

The dimension of the equilibrium manifold is:

$$\lambda = 5 - 3 = 2.$$

The network is, as in the previous case, under-dimensioned. There are two mass conservation laws, with matrix B given by:

$$B^T = \begin{pmatrix} 1 & 0 & 1 & 1 & 0 \\ 0 & 1 & 1 & 0 & 1 \end{pmatrix}.$$

The matrix Λ associated to the linkage classes reads:

$$\Lambda^T = \begin{pmatrix} 1 & 0 & 0 & 1 & 0 & 0 & 0 \\ 0 & 1 & 0 & 0 & 1 & 0 & 0 \\ 0 & 0 & 1 & 0 & 0 & 1 & 1 \end{pmatrix}.$$

Starting from the stoichiometric matrix Y and the matrix Λ we compute a basis for the deficiency subspace as:

$$\omega = \begin{pmatrix} 1 & -1 & 0 & -1 & 1 & 1 & -1 \end{pmatrix}^T,$$

and, therefore, we can express $A\psi$ as:

$$\begin{aligned} -k_{14}\psi_1 + k_{41}\psi_4 &= \alpha_1 \\ -k_{25}\psi_2 + k_{52}\psi_5 &= -\alpha_1 \\ k_{36}\psi_3 - k_{63}\psi_6 &= \alpha_1 \\ k_{37}\psi_3 - k_{73}\psi_7 &= -\alpha_1. \end{aligned}$$

Substituting the elements of vector ψ by the concentration monomials we obtain the equations defining the locus of equilibria:

$$\begin{aligned} c_4 - k_{14}/k_{41}c_1 - \alpha_1/k_{41} &= 0 \\ c_5 - k_{25}/k_{52}c_2 + \alpha_1/k_{52} &= 0 \\ -c_2c_4 + k_{36}/k_{63}c_3 - \alpha_1/k_{63} &= 0 \\ -c_1c_5 + k_{37}/k_{73}c_3 + \alpha_1/k_{73} &= 0. \end{aligned}$$

Then we can express the concentrations as:

$$\begin{aligned} c_1 &= (k_{41}c_4 - \alpha_1)/k_{41} \\ c_2 &= -\alpha_1(1 + k_{37}/k_{36} + k_{73}/k_{52}c_1)/(k_{37}k_{63}/k_{36}c_4 - k_{73}/k_{52}k_{25}c_1) \\ c_3 &= (k_{63}c_2c_4 + \alpha_1)/k_{36} \\ c_5 &= (k_{25}c_2 - \alpha_1)/k_{52}. \end{aligned}$$

From Eq. (12) in the main text we get the matrix G:

$$G = \begin{pmatrix} -k_{14}/k_{41} & 0 & 0 & 1 & 0 & -1/k_{41} \\ 0 & -k_{25}/k_{52} & 0 & 0 & 1 & 1/k_{52} \\ 0 & -c_4 & k_{36}/k_{63} & -c_2 & 0 & -1/k_{63} \\ -c_5 & 0 & k_{37}/k_{73} & 0 & -c_1 & 1/k_{73} \\ 1 & 0 & 1 & 1 & 0 & 0 \\ 0 & 1 & 1 & 0 & 1 & 0 \end{pmatrix}.$$

We formulate an optimization problem to find parameter vectors for which the determinant of G vanishes. The objective function is $F_{def} = det(G)^2$ and we use the following decision vector:

$$\text{x} = (k_{41},\ k_{14},\ k_{25},\ k_{52},\ k_{63},\ k_{36},\ k_{37},\ k_{73},\ \alpha_1, c_4).$$

We solve the optimization problem (Eq. 14 in the main text) subject to positive concentration vectors (and the equilibrium manifold equations) using the global scatter search solver by Egea et al. [7]. No parameter vector was found for which the objective function becomes zero. The Matlab code is provided in S1 file (`Matlab_code/IFN_receptor/Variant2`).

4.3 Semi-diffusive network with constant IFN inflow

The system in Fig. 3B, taking into account all the complexes and fluxes, including the inflow and outflow fluxes indicated by dashed arrows, is a semi-diffusive network. The molecularity matrix Y containing the molecularity of

each species in each complex is given by Eq. (4.1). The matrix containing the molecularities of the species in the source complexes for true and outflow reactions of the semi-diffusive network is:

$$Y_{to} = \begin{pmatrix} 0 & 0 & 0 & 1 & 1 & 0 & 0 & 0 & 1 & 0 & 0 \\ 0 & 0 & 0 & 1 & 0 & 0 & 1 & 0 & 0 & 1 & 0 \\ 0 & 0 & 0 & 0 & 1 & 1 & 0 & 0 & 0 & 0 & 1 \\ 1 & 0 & 0 & 0 & 0 & 1 & 0 & 0 & 0 & 0 & 0 \\ 0 & 1 & 0 & 0 & 0 & 0 & 1 & 0 & 0 & 0 & 0 \\ 0 & 0 & 1 & 0 & 0 & 0 & 0 & 0 & 0 & 0 & 0 \end{pmatrix},$$

and the corresponding stoichiometric matrix can be computed starting from the stoichiometric matrix of the true reaction subnetwork (closed system in Section 1.1) as:

$$S_{to} = \left[S \,\middle|\, -I_{6\times 6} \right].$$

There are in total 14 fluxes corresponding to true and outflow reactions (we denote the flux vector by μ). From Eq. (17) in the main text we have that:

$$p(c, k) = -S_{to} v_{to}(c, k),$$

and in terms of the fluxes:

$$\overline{p}(\mu) = -S_{to}\mu. \tag{4.3}$$

The vector containing the basal formation rates of the key species (I, $R1$ and $R2$) is:

$$K = \begin{pmatrix} v_{15} & v_{16} & v_{17} & 0 & 0 & 0 \end{pmatrix}^T,$$

where $v_{15} = k_{15}$, $v_{16} = k_{16}$ and $v_{17} = k_{17}$ are the (constant) reaction rates of the reactions r_{15}, r_{16} and r_{17}.

Let us build a matrix Y_r with columns corresponding to the source complexes for the true and outlet reactions, such as:

$$Y_r = \left[Y_4 \quad Y_1 \quad Y_2 \quad Y_5 \quad Y_6 \quad Y_3 \quad Y_3 \quad Y_7 \,\middle|\, I_{6\times 6} \right].$$

Now, we are ready to define the objective function $F_{inj} = S_{to} diag(\mu) Y_r^T$ to minimize, searching values of the decision vector of fluxes μ that make it vanish. The search is subject, on the one hand, to the following inequality constraints (corresponding to the key species I, $R1$, $R2$):

$$\begin{aligned} \mu_1 - \mu_2 - \mu_3 + \mu_4 + \mu_9 &> 0 \\ \mu_1 - \mu_2 - \mu_7 + \mu_8 + \mu_{10} &> 0 \\ -\mu_3 + \mu_4 + \mu_5 - \mu_6 + \mu_{11} &> 0, \end{aligned}$$

where the expressions at the left hand side of the inequalities are given by $p(1)$, $p(2)$ and $p(3)$ in (4.3) and, on the other hand, to the following equality constraints (corresponding to the remaining species):

$$\begin{aligned}-\mu_1 + \mu_2 + \mu_5 - \mu_6 + \mu_{12} = 0\\ +\mu_3 - \mu_4 - \mu_7 + \mu_8 + \mu_{13} = 0\\ -\mu_5 + \mu_6 + \mu_7 - \mu_8 + \mu_{14} = 0,\end{aligned}$$

where the expressions at the left hand side are given by $p(4)$, $p(5)$ and $p(6)$ in (4.3). We solve the optimization problem[1] (Eq. 18 in the main text) using the global scatter search solver by Egea et al. [7], and no positive vector of fluxes was found for which the objective function becomes zero. The Matlab code is provided in S1 file (`Matlab_code/IFN_receptor/Variant3`).

4.4 Semi-diffusive network with IFN in excess

The network in Fig. 3C, taking into account all the complexes and fluxes (including the inflow and outflow fluxes indicated by dashed arrows) is a semi-diffusive network. The molecularity matrix Y containing the molecularity of each species in each complex is given by Eq. (4.2). The matrix containing the molecularities of the species in the source complexes for true and outflow reactions of the semi-diffusive network is:

$$Y_{to} = \begin{pmatrix} 1 & 0 & 0 & 0 & 0 & 0 & 1 & 0 \\ 0 & 1 & 0 & 0 & 0 & 1 & 0 & 0 \\ 0 & 0 & 1 & 0 & 0 & 0 & 0 & 0 \\ 0 & 0 & 0 & 1 & 0 & 1 & 0 & 0 \\ 0 & 0 & 0 & 0 & 1 & 0 & 1 & 0 \end{pmatrix},$$

and the stoichiometric matrix can be computed starting from the molecularity matrix of its true-reaction network counterpart (closed network in Section 1.2):

$$S_{to} = \left[S \,\middle|\, -I_{5\times5} \right].$$

There are in total 13 fluxes corresponding to true and outflow reactions (contained in the vector μ). From Eq. (17) in the main text we have that:

$$p(c, k) = -S_{to} v_{to}(c, k),$$

and in terms of the fluxes:

$$\overline{p}(\mu) = -S_{to}\mu. \qquad (4.4)$$

[1]Note that the equality constraints can be used to express some fluxes in terms others and reduce the number of decision variables.

The vector of basal formation rates of the key species, $R1$ and $R2$, is:

$$K = \begin{pmatrix} v_{14} & v_{15} & 0 & 0 & 0 \end{pmatrix}^T,$$

where $v_{14} = k_{14}$ and $v_{15} = k_{15}$ are the (constant) reaction rates of the reactions r_{14} and r_{15}. We build a matrix Y_r with columns corresponding to the source complexes of the true and outlet reactions:

$$Y_r = \left[Y_4 \quad Y_1 \quad Y_2 \quad Y_5 \quad Y_6 \quad Y_3 \quad Y_3 \quad Y_7 \,\middle|\, I_{5\times 5} \right].$$

We define now the objective function $F_{inj} = S_{to} diag(\mu) Y_r^T$ and search for values of the decision vector of fluxes μ that make it vanish. The search is subject, on the one hand, to the following inequality constraints (corresponding to the key species $R1$ and $R2$):

$$\begin{aligned} -\mu_1 + \mu_2 - \mu_7 + \mu_8 + \mu_9 &> 0 \\ \mu_3 - \mu_4 + \mu_5 - \mu_6 + \mu_{10} &> 0 \end{aligned}$$

where the expressions at the left hand side are given by $p(1)$ and $p(2)$ in (4.4) and, on the other hand, to the following equality constraints:

$$\begin{aligned} -\mu_5 + \mu_6 + \mu_7 - \mu_8 + \mu_{11} &= 0 \\ \mu_1 - \mu_2 + \mu_5 - \mu_6 + \mu_{12} &= 0 \\ -\mu_3 + \mu_4 - \mu_7 + \mu_8 + \mu_{13} &= 0 \end{aligned}$$

where the expressions at the left hand side are given by $p(3)$, $p(4)$ and $p(5)$ in (4.4). We solve the optimization problem[2] (Eq. 18 in the main text) using the global scatter search solver by Egea et al. [7]. No positive vector of fluxes was found for which the objective function becomes zero. The Matlab code is provided in S1 file (`Matlab_code/IFN_receptor/Variant4`).

[2]Note that the equality constraints can be used to express some fluxes in terms others and reduce the number of decision variables.

Chapter 5

Early STAT signaling upon interferon stimulation

5.1 Closed network

There are $N = 9$ species participating in the network with concentrations $c_1 = [R^*]$ (activated receptor complex), $c_2 = [S1]$, $c_3 = [S2]$, $c_4 = [S1^*]$, $c_5 = [S2^*]$, $c_6 = [R^*S2^*]$, $c_7 = [R^*S2^*S1^*]$, $c_8 = [S1^*S1^*]$ and $c_9 = [S1^*S2^*]$. The complexes are labeled such that $\mathcal{C}_1 = R^*S2^*$, $\mathcal{C}_2 = R^*S2^*S1^*$, $\mathcal{C}_3 = S1^*S1^*$, $\mathcal{C}_4 = S1^*S2^*$, $\mathcal{C}_5 = S1^* + S2^*$, $\mathcal{C}_6 = S1 + S1$, $\mathcal{C}_7 = R^* + S2$, $\mathcal{C}_8 = R^* + S2^*$, $\mathcal{C}_9 = R^*S2^* + S1^*$, $\mathcal{C}_{10} = R^*S2^* + S1$, $\mathcal{C}_{11} = S1^* + S1^*$ and $\mathcal{C}_{12} = S1 + S2$, $(M = 12)$.

The molecularity matrix is:

$$Y = \begin{pmatrix} 0 & 0 & 0 & 0 & 0 & 0 & 1 & 1 & 0 & 0 & 0 & 0 \\ 0 & 0 & 0 & 0 & 0 & 2 & 0 & 0 & 0 & 1 & 0 & 1 \\ 0 & 0 & 0 & 0 & 0 & 0 & 1 & 0 & 0 & 0 & 0 & 1 \\ 0 & 0 & 0 & 0 & 1 & 0 & 0 & 0 & 1 & 0 & 2 & 0 \\ 0 & 0 & 0 & 0 & 1 & 0 & 0 & 1 & 0 & 0 & 0 & 0 \\ 1 & 0 & 0 & 0 & 0 & 0 & 0 & 0 & 1 & 1 & 0 & 0 \\ 0 & 1 & 0 & 0 & 0 & 0 & 0 & 0 & 0 & 0 & 0 & 0 \\ 0 & 0 & 1 & 0 & 0 & 0 & 0 & 0 & 0 & 0 & 0 & 0 \\ 0 & 0 & 0 & 1 & 0 & 0 & 0 & 0 & 0 & 0 & 0 & 0 \end{pmatrix} \tag{5.1}$$

and taking mass action kinetics, the vector of mass action monomials reads:

$$\psi(c) = \begin{pmatrix} c_6, & c_7, & c_8, & c_9, & c_4c_5, & c_2^2, & c_1c_3, & c_1c_5, & c_4c_6, & c_2c_6, & c_4^2, & c_2c_3 \end{pmatrix}^T.$$

The stoichiometric matrix is:

$$S = \begin{pmatrix} -1 & 1 & 1 & 0 & 0 & 0 & 0 & 0 & 0 \\ 0 & 0 & 0 & -1 & 0 & 0 & 2 & 0 & 1 \\ -1 & 1 & 0 & 0 & 0 & 0 & 0 & 0 & 1 \\ 0 & 0 & 0 & 0 & 1 & -2 & 0 & -1 & 0 \\ 0 & 0 & 1 & 0 & 0 & 0 & 0 & -1 & 0 \\ 1 & -1 & -1 & -1 & 1 & 0 & 0 & 0 & 0 \\ 0 & 0 & 0 & 1 & -1 & 0 & 0 & 0 & 0 \\ 0 & 0 & 0 & 0 & 0 & 1 & -1 & 0 & 0 \\ 0 & 0 & 0 & 0 & 0 & 0 & 0 & 1 & -1 \end{pmatrix}.$$

The rank of the matrix S (and, therefore, the dimension of the stoichiometric subspace) is $s = 6$. The deficiency of the network is:

$$\delta = 12 - 4 - 6 = 2,$$

and the dimension of the equilibrium manifold:

$$\lambda = 9 - 6 = 3.$$

The network is under-dimensioned ($\lambda > \delta$). There are three mass conservation laws defined by $W(c, \sigma) = 0$, where $W(c, \sigma) = B^T c - \sigma$, $\sigma = B^T c$, and

$$B^T = \begin{pmatrix} 1 & 0 & 0 & 0 & 0 & 1 & 1 & 0 & 0 \\ 0 & 1 & 0 & 1 & 0 & 0 & 1 & 2 & 1 \\ 0 & 0 & 1 & 0 & 1 & 1 & 1 & 0 & 1 \end{pmatrix}.$$

Note that the strictly positive vector $\vartheta = (1, 1, 1, 1, 1, 2, 3, 2, 2)$ fulfills $\vartheta S = 0$, as it corresponds to a closed network. The matrix Λ associated to the linkage classes reads:

$$\Lambda^T = \begin{pmatrix} 1 & 0 & 0 & 0 & 0 & 0 & 1 & 1 & 0 & 0 & 0 & 0 \\ 0 & 1 & 0 & 0 & 0 & 0 & 0 & 0 & 1 & 1 & 0 & 0 \\ 0 & 0 & 1 & 0 & 0 & 1 & 0 & 0 & 0 & 0 & 1 & 0 \\ 0 & 0 & 0 & 1 & 1 & 0 & 0 & 0 & 0 & 0 & 0 & 1 \end{pmatrix}.$$

Starting from the stoichiometric matrix Y and the matrix Λ we compute a basis for the deficiency subspace as:

$$\omega = \begin{pmatrix} 0 & 0 & 0 & 0 & 0 & -1 & 0 & 0 & -2 & 2 & 1 & 0 \\ 0 & 0 & 0 & 0 & -1 & 0 & -1 & 1 & 1 & -1 & 0 & 1 \end{pmatrix}^T,$$

and, therefore, we can express $A\psi$ as:

$$\begin{aligned}
-k_{5\,4}\psi_5 &= -\alpha_2 \\
k_{3\,6}\psi_3 &= -\alpha_1 \\
k_{1\,7}\psi_1 - k_{7\,1}\psi_7 &= -\alpha_2 \\
k_{1\,8}\psi_1 &= \alpha_2 \\
k_{2\,9}\psi_2 &= -2\alpha_1 + \alpha_2 \\
-k_{10\,2}\psi_{10} &= 2\alpha_1 - \alpha_2 \\
-k_{11\,3}\psi_{11} &= \alpha_1 \\
k_{4\,12}\psi_4 &= \alpha_2.
\end{aligned}$$

Substituting the elements of vector ψ by the concentration monomials we obtain the equations defining the locus of equilibria:

$$\begin{aligned}
c_4c_5 - \alpha_2/k_{5\,4} &= 0 \\
c_8 + \alpha_1/k_{3\,6} &= 0 \\
k_{1\,7}/k_{7\,1}c_6 - c_1c_3 + \alpha_2/k_{7\,1} &= 0 \\
c_6 - \alpha_2/k_{1\,8} &= 0 \\
c_7 + 2\alpha_1/k_{2\,9} - \alpha_2/k_{2\,9} &= 0 \\
-c_2c_6 - 2\alpha_1/k_{10\,2} + \alpha_2/k_{10\,2} &= 0 \\
c_4^2 + \alpha_1/k_{11\,3} &= 0 \\
c_9 - \alpha_2/k_{4\,12} &= 0.
\end{aligned}$$

We can express the concentrations as:

$$\begin{aligned}
c_6 &= \alpha_2/k_{1\,8} \\
c_7 &= -2\alpha_1/k_{2\,9} + \alpha_2/k_{2\,9} \\
c_8 &= -\alpha_1/k_{3\,6} \\
c_9 &= \alpha_2/k_{4\,12} \\
c_2 &= (-2\alpha_1/k_{10\,2} + \alpha_2/k_{10\,2})/c_6 \\
c_4 &= (-\alpha_1/k_{11\,3})^{1/2} \\
c_5 &= \alpha_2 c_4/k_{5\,4} \\
c_3 &= (\alpha_2/k_{7\,1} + k_{1\,7}c_6/k_{7\,1})/c_1.
\end{aligned}$$

The matrix G, from Eq. (13) in the main text, reads:

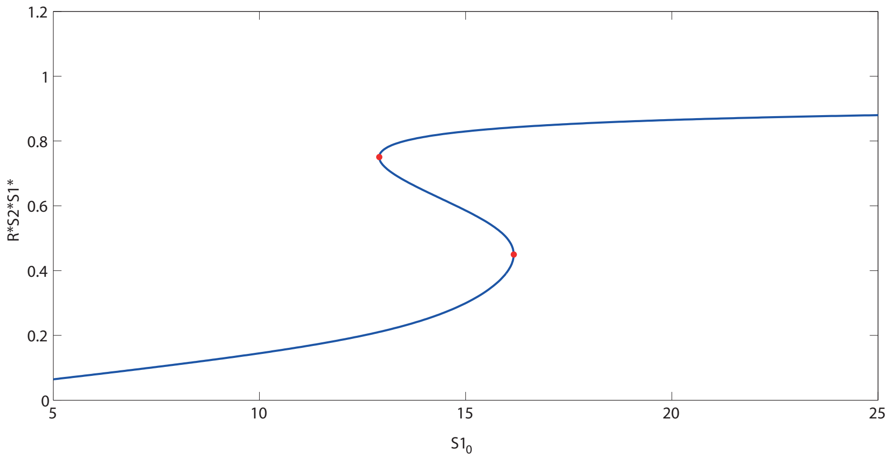

Figure 5.1: Bifurcation diagram for the STAT network. A continuation of equilibria is started from the optimal point found by our algorithm (in forward and backward directions). Tangent bifurcation points are indicated in red.

$$G = \begin{pmatrix} 0 & 0 & 0 & c_5 & c_4 & 0 & 0 & 0 & 0 & 0 & -1/k_{5\,4} \\ 0 & 0 & 0 & 0 & 0 & 0 & 0 & 1 & 0 & 1/k_{3\,6} & 0 \\ -c_3 & 0 & -c_1 & 0 & 0 & k_{1\,7}/k_{7\,1} & 0 & 0 & 0 & 0 & 1/k_{7\,1} \\ 0 & 0 & 0 & 0 & 0 & 1 & 0 & 0 & 0 & 0 & -1/k_{1\,8} \\ 0 & 0 & 0 & 0 & 0 & 0 & 1 & 0 & 0 & 2/k_{2\,9} & -1/k_{2\,9} \\ 0 & -c_6 & 0 & 0 & 0 & -c_2 & 0 & 0 & 0 & -2/k_{10\,2} & 1/k_{10\,2} \\ 0 & 0 & 0 & 2c_4 & 0 & 0 & 0 & 0 & 0 & 1/k_{11\,3} & 0 \\ 0 & 0 & 0 & 0 & 0 & 0 & 0 & 0 & 1 & 0 & -1/k_{4\,12} \\ 1 & 0 & 0 & 0 & 0 & 1 & 1 & 0 & 0 & 0 & 0 \\ 0 & 1 & 0 & 1 & 0 & 0 & 1 & 2 & 1 & 0 & 0 \\ 0 & 0 & 1 & 0 & 1 & 1 & 1 & 0 & 1 & 0 & 0 \end{pmatrix}.$$

We have that $N + \delta = 11$, which gives us the dimensions of the matrix G (11×11). We formulate the optimization problem defined by Eq. (14) in the main text, to find parameter vectors for which the determinant of matrix G becomes zero. We minimize the objective function $F_{def} = det(G)^2$ using as decision vector:

$$\text{x} = (k_{71},\ k_{17},\ k_{18},\ k_{10\,2},\ k_{2\,9},\ k_{11\,3},\ k_{3\,6},\ k_{5\,4},\ k_{4\,12}, \alpha_1, \alpha_2, c_1).$$

We solve the optimization problem subject to positive concentration vectors (and the equilibrium manifold equations) using the global scatter search solver by Egea et al. [7]. We found a decision vector for which the objective function vanishes (see Table 5.1). At this point, the system undergoes a saddle-node bifurcation. We start a bifurcation analysis from this steady

state, confirming that in fact the network has capacity for bistability (see Fig 5.1). The Matlab code is provided in S1 file (`Matlab_code/STATS_early/Variant1`).

Table 5.1: A set of parameters leading to bistability for the (closed) network in Fig. 5A

k_{71}	k_{17}	k_{18}	$k_{10\,2}$	k_{29}	$k_{11\,3}$	k_{36}	k_{54}	$k_{4\,12}$	α_1	α_2	c_1
94.510	0.100	55.1276	99.168	100	74.626	99.999	15.225	1.457	-10.077	21.984	0.103

5.2 Semi-diffusive network

We consider the network in Fig. 5A taking into account all the fluxes (including the inflow and outflow fluxes represented by dashed arrows). The molecularity matrix is given by Eq. (5.1). We build the stoichiometric matrix of the true and outflow reactions starting from matrix S for the true reaction subnetwork (closed network in section 2.1) as:

$$S_{to} = \left[S \,\middle|\, -I_{9\times9} \right].$$

There are in total 18 fluxes corresponding to true and outflow reactions (contained in the vector μ).

From Eq. (17) in the main text we have that:

$$p(c,k) = -S_{to}v_{to}(c,k),$$

and in terms of the fluxes:

$$\overline{p}(\mu) = -S_{to}\mu. \tag{5.2}$$

The vector of basal formation rates of the key species (R^*, $S1$ and $S2$) is:

$$K = \begin{pmatrix} v_{19} & v_{20} & v_{21} & 0 & 0 & 0 & 0 & 0 & 0 \end{pmatrix}^T,$$

where $v_{19} = k_{19}$, $v_{20} = k_{20}$ and $v_{21} = k_{21}$ are the (constant) reaction rates of the reactions r_{19} to r_{21}. We build a matrix Y_r with columns corresponding to the source complexes of the true and outlet reactions:

$$Y_r = \left[Y_7 \quad Y_1 \quad Y_1 \quad Y_{10} \quad Y_2 \quad Y_{11} \quad Y_3 \quad Y_5 \quad Y_4 \,\middle|\, I_{9\times9} \right].$$

We can now define the objective function $F_{inj} = S_{to}diag(\mu)Y_r^T$, and search for values of the decision vector of fluxes μ that make it vanish. The search

is subject, on the one hand, to the following inequality constraints (corresponding to the key species):

$$\begin{aligned}
\mu_1 - \mu_2 - \mu_3 + \mu_{10} &> 0 \\
\mu_4 - 2\mu_7 - \mu_9 + \mu_{11} &> 0 \\
\mu_1 - \mu_2 - \mu_9 + \mu_{12} &> 0
\end{aligned}$$

where the expressions at the left hand side are given by $p(1)$, $p(2)$ and $p(3)$ in Eq. (5.2) and, on the other hand, to the following equality constraints:

$$\begin{aligned}
-\mu_5 + 2\mu_6 + \mu_8 + \mu_{13} &= 0 \\
-\mu_3 + \mu_8 + \mu_{14} &= 0 \\
-\mu_1 + \mu_2 + \mu_3 + \mu_4 - \mu_5 + \mu_{15} &= 0 \\
-\mu_4 + \mu_5 + \mu_{16} &= 0 \\
-\mu_6 + \mu_7 + \mu_{17} &= 0 \\
-\mu_8 + \mu_9 + \mu_{18} &= 0
\end{aligned}$$

where the expressions at the left hand side are given by $p(4)$ to $p(6)$ in Eq. (5.2). We solve the optimization problem[1] (Eq. 18 in the main text) using the global scatter search solver by Egea et al. [7]. We found a decision vector for which the objective function vanishes[2] (see Table 5.2). We now compute a steady state concentration vector and a set of kinetic parameters compatible with these fluxes. We fix the values of the concentrations such that $c = 1$, and the kinetic rate constants remain equal to the fluxes, i.e., $k_i = \mu_i$ for $i = 1, \ldots, 18$. Starting a continuation of equilibria from this point, we confirm that the system undergoes a saddle-node bifurcation, and that it has capacity for bistability (see Fig 5.2). The Matlab code is provided in S1 file (`Matlab_code/STATS_early/Variant2`).

Table 5.2: A set of fluxes leading to bistability for the (open) network in Fig. 5A

μ_1	μ_2	μ_3	μ_4	μ_5	μ_6	μ_7	μ_8	μ_9
99.995	0.022	39.899	99.943	39.919	0.010	0.010	39.899	0.010

μ_{10}	μ_{11}	μ_{12}	μ_{13}	μ_{14}	μ_{15}	μ_{16}	μ_{17}	μ_{18}
99.951	0.011	0.924	0.0001	4.41×1^{-5}	0.05	60.0234	6.32×1^{-6}	39.8894

[1]Note that the equality constraints can be used to express some fluxes in terms of others reducing the number of decision variables.

[2]For numerical reasons, a small determinant close to zero might not result in a zero eigenvalue of the Jacobian. We check whether the Jacobian has a zero eigenvalue leading to a saddle-node bifurcation by performing a bifurcation analysis.

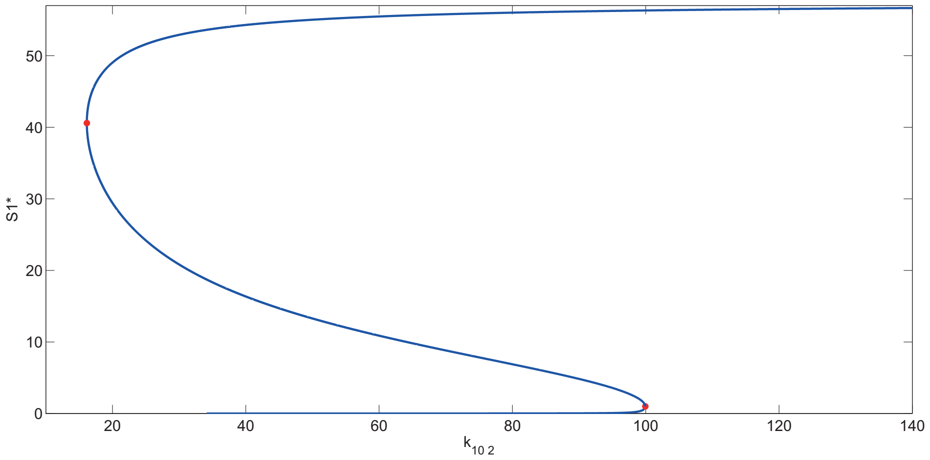

Figure 5.2: Bifurcation diagram for the early STAT open network. A continuation of equilibria is started from the optimal point found by our algorithm (in forward and backward directions). Tangent bifurcation points are indicated in red.

Chapter 6

STAT signaling and feedback via STAT1 expression

We consider the network in Fig. 5B, taking into account all the reactions (including the inflow and outflow reactions represented by dashed lines). The network is semi-diffusive. There are twelve species participating in the network with concentrations $c_1 = [R^*]$ (activated receptor complex), $c_2 = [S1]$, $c_3 = [S2]$, $c_4 = [S1^*]$, $c_5 = [S2^*]$, $c_6 = [R^*S2^*]$, $c_7 = [R^*S2^*S1^*]$, $c_8 = [S1^*S1^*]$, $c_9 = [S1^*S2^*]$, $c_{10} = [IRF1]$, $c_{11} = [CBP]$, $c_{12} = [IRF1CBP]$. The complexes are labelled such that $\mathcal{C}_1 = R^*S2^*$, $\mathcal{C}_2 = R^*S2^*S1^*$, $\mathcal{C}_3 = S1^*S1^*$, $\mathcal{C}_4 = S1^*S2^*$, $\mathcal{C}_5 = IRF1CBP$, $\mathcal{C}_6 = S1 + S1$, $\mathcal{C}_7 = R^* + S2$, $\mathcal{C}_8 = R^* + S2^*$, $\mathcal{C}_9 = R^*S2^* + S1^*$, $\mathcal{C}_{10} = R^*S2^* + S1$, $\mathcal{C}_{11} = S1^* + S1^*$, $\mathcal{C}_{12} = S1 + S2$, $\mathcal{C}_{13} = S1^*S1^* + IRF1$, $\mathcal{C}_{14} = S1^* + S2^*$, $\mathcal{C}_{15} = IRF1 + CBP$, $\mathcal{C}_{16} = IRF1CBP + S1$, $\mathcal{C}_{17} = \emptyset$, $\mathcal{C}_{18} = R^*$, $\mathcal{C}_{19} = S1$, $\mathcal{C}_{20} = S2$, $\mathcal{C}_{21} = S1^*$, $\mathcal{C}_{22} = S2^*$, $\mathcal{C}_{23} = IRF1$, $\mathcal{C}_{24} = CBP$. The molecularity matrix corresponding to the true reaction subnetwork (without inflow and outflow

reactions) is:

$$Y = \begin{pmatrix} 0&0&0&0&0&0&1&1&0&0&0&0&0&0&0&0 \\ 0&0&0&0&0&2&0&0&0&1&0&1&0&0&0&1 \\ 0&0&0&0&0&0&1&0&0&0&0&1&0&0&0&0 \\ 0&0&0&0&0&0&0&0&1&0&2&0&0&1&0&0 \\ 0&0&0&0&0&0&0&1&0&0&0&0&0&1&0&0 \\ 1&0&0&0&0&0&0&0&1&1&0&0&0&0&0&0 \\ 0&1&0&0&0&0&0&0&0&0&0&0&0&0&0&0 \\ 0&0&1&0&0&0&0&0&0&0&0&0&1&0&0&0 \\ 0&0&0&1&0&0&0&0&0&0&0&0&0&0&0&0 \\ 0&0&0&0&0&0&0&0&0&0&0&0&1&0&1&0 \\ 0&0&0&0&0&0&0&0&0&0&0&0&0&0&1&0 \\ 0&0&0&0&1&0&0&0&0&0&0&0&0&0&0&1 \end{pmatrix}$$

and the stoichiometric matrix:

$S = \left[\; Y_1 - Y_7, Y_7 - Y_1, Y_8 - Y_1, Y_2 - Y_{10}, Y_9 - Y_2, Y_3 - Y_{11}, Y_6 - Y_3, Y_4 - Y_{14}, Y_{12} - Y_4, Y_5 - Y_{15}, Y_{16} - Y_5, Y_{13} - Y_3 \;\right].$

The stoichiometric matrix of the true and outflow reactions is:

$$S_{to} = \left[S \,\middle|\, -I_{12\times 12} \right]$$

There are in total 24 fluxes corresponding to true and outflow reactions (contained in the vector μ).

From Eq. (17) in the main text we have that:

$$p(c,k) = -S_{to} v_{to}(c,k),$$

and in terms of the fluxes:

$$\overline{p}(\mu) = -S_{to}\mu. \tag{6.1}$$

The vector of basal formation rates of the key species, R^*, $S1$, $S2$, $IRF1$ and CBP is:

$$K = (v_{25} \;\; v_{26} \;\; v_{27} \;\; v_{28} \;\; v_{29} \;\; 0 \;\; 0 \;\; 0 \;\; 0 \;\; 0 \;\; 0 \;\; 0)^T$$

where $v_{25} = k_{25}$, $v_{26} = k_{26}$, $v_{27} = k_{27}$, $v_{28} = k_{28}$ and $v_{29} = k_{29}$ are the (constant) reaction rates of the reactions r_{25} to r_{29}. We build a matrix Y_r with columns corresponding to the source complexes of the true and outlet reactions:

$$Y_r = [Y_7 \;\; Y_1 \;\; Y_1 \;\; Y_{10} \;\; Y_2 \;\; Y_{11} \;\; Y_3 \;\; Y_{14} \;\; Y_4 \;\; Y_{15} \;\; Y_5 \;\; Y_3 | \;\; I_{12\times 12}].$$

We can define now the objective function $F_{inj} = S_{to} diag(\mu) Y_r^T$, and search for values of the decision vector of fluxes μ that make it vanish. The search

is subject, on the one hand, to the following inequality constraints (corresponding to the key species):

$$\begin{aligned}\mu_1 - \mu_2 - \mu_3 + \mu_{13} &> 0\\ \mu_4 - 2\mu_7 - \mu_9 - \mu_{11} + \mu_{14} &> 0\\ \mu_1 - \mu_2 - \mu_9 + \mu_{15} &> 0\\ \mu_{10} - \mu_{12} + \mu_{22} &> 0\\ \mu_{10} + \mu_{23} &> 0\end{aligned}$$

where the expressions at the left hand side are obtained from $p(1)$, $p(2)$, $p(3)$, $p(10)$ and $p(11)$ and, on the other hand, to the equality constraints:

$$\begin{aligned}-\mu_5 + 2\mu_6 + \mu_8 + \mu_{16} &= 0\\ -\mu_3 + \mu_8 + \mu_{17} &= 0\\ -\mu_1 + \mu_2 + \mu_3 + \mu_4 - \mu_5 + \mu_{18} &= 0\\ -\mu_4 + \mu_5 + \mu_{19} &= 0\\ -\mu_6 + \mu_7 + \mu_{20} &= 0\\ -\mu_8 + \mu_9 + \mu_{21} &= 0\\ -\mu_{10} + \mu_{24} &= 0\end{aligned}$$

where the expressions at the left hand side are obtained from $p(4)$, $p(5)$, $p(6)$, $p(7)$, $p(8)$, $p(9)$ and $p(12)$. We solve the optimization problem[1] (Eq. 18 in the main text) using the global scatter search solver by Egea et al. [7]. We found a decision vector for which the objective function vanishes (see Table 6.1). We now compute a steady state concentration vector and a set of kinetic parameters compatible with these fluxes. We fix the values of the concentrations such that $c = 1$, and the kinetic rate constants remain equal to the fluxes, i.e., $k_i = \mu_i$ for $i = 1, \ldots, 24$. Starting a continuation of equilibria from this point, we confirm that the system undergoes a saddle-node bifurcation, and that it has capacity for bistability (see Fig 6.1).

The Matlab code is provided in S1 file (`Matlab_code/STATS_feedback`).

[1] Note that the equality constraints can be used to express some fluxes in terms others and reduce the number of decision variables.

Table 6.1: Optimum set of fluxes for the (open) network in Fig. 5B.

μ_1	μ_2	μ_3	μ_4	μ_5	μ_6	μ_7	μ_8
4.9524	0.0283	4.8871	4.5933	4.5831	0.0259	0.0141	4.2792

μ_9	μ_{10}	μ_{11}	μ_{12}	μ_{13}	μ_{14}	μ_{15}	μ_{16}
2.1459	0.0101	4.9812	0.01828	9.6457	43.9942	3.7295	0.2521

μ_{17}	μ_{18}	μ_{19}	μ_{20}	μ_{21}	μ_{22}	μ_{23}	μ_{24}
0.6079	0.0268	0.0101	0.0118	2.1332	0.0100	2.7615	0.0101

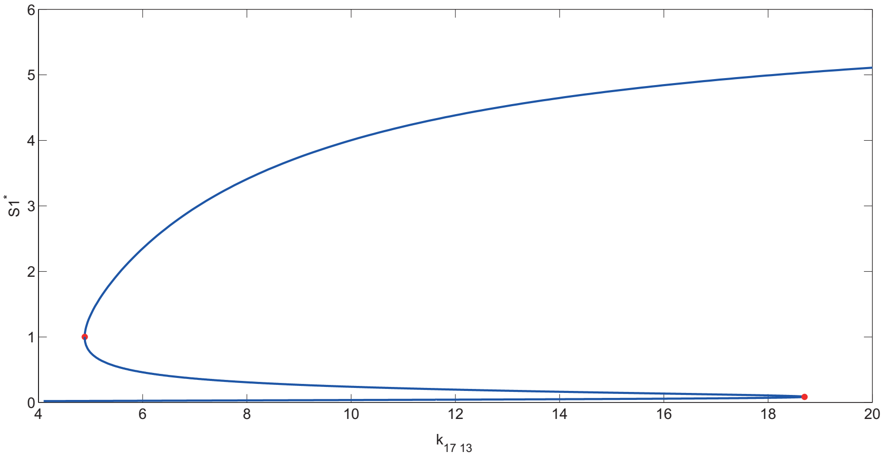

Figure 6.1: Bifurcation diagram for the STAT signaling network with feedback via STAT1 expression. A continuation of equilibria is started from a steady state compatible with the optimal point found by our algorithm (in forward and backward directions). Tangent bifurcation points are indicated in red.

Chapter 7

Receptor complex formation and STAT signaling

There are 13 species participating in the network with concentrations $c_1 = [R1]$, $c_2 = [R2]$, $c_3 = [R^*]$ (denoting activated receptor complex), $c_4 = [R1I]$, $c_5 = [R2I]$, $c_6 = [S1]$, $c_7 = [S2]$, $c_8 = [S1^*]$, $c_9 = [S2^*]$, $c_{10} = [R^*S2^*]$, $c_{11} = [R^*S2^*S1^*]$, $c_{12} = [S1^*S1^*]$, $c_{13} = [S1^*S2^*]$. The complexes are labelled such that $\mathcal{C}_1 = R1$, $\mathcal{C}_2 = R2$, $\mathcal{C}_3 = R^*$, $\mathcal{C}_4 = R^*S2^*$, $\mathcal{C}_5 = R^*S2^*S1^*$, $\mathcal{C}_6 = S1^*S1^*$, $\mathcal{C}_7 = S1^*S2^*$, $\mathcal{C}_8 = R1I$, $\mathcal{C}_9 = R2I$, $\mathcal{C}_{10} = R2 + R1I$, $\mathcal{C}_{11} = R1{+}R2I$, $\mathcal{C}_{12} = R^*{+}S2$, $\mathcal{C}_{13} = R^*{+}S2^*$, $\mathcal{C}_{14} = R^*S2^*{+}S1$, $\mathcal{C}_{15} = R^*S2^*S1^*$, $\mathcal{C}_{16} = S1^* + S1^*$, $\mathcal{C}_{17} = S1 + S1$, $\mathcal{C}_{18} = S1^* + S2^*$ and $\mathcal{C}_{19} = S1 + S2$.

The molecularity matrix is:

$$Y = \begin{pmatrix}
1 & 0 & 0 & 0 & 0 & 0 & 0 & 0 & 0 & 0 & 1 & 0 & 0 & 0 & 0 & 0 & 0 & 0 & 0 \\
0 & 1 & 0 & 0 & 0 & 0 & 0 & 0 & 0 & 1 & 0 & 0 & 0 & 0 & 0 & 0 & 0 & 0 & 0 \\
0 & 0 & 1 & 0 & 0 & 0 & 0 & 0 & 0 & 0 & 0 & 1 & 1 & 0 & 0 & 0 & 0 & 0 & 0 \\
0 & 0 & 0 & 0 & 0 & 0 & 0 & 1 & 0 & 1 & 0 & 0 & 0 & 0 & 0 & 0 & 0 & 0 & 0 \\
0 & 0 & 0 & 0 & 0 & 0 & 0 & 0 & 1 & 0 & 1 & 0 & 0 & 0 & 0 & 0 & 0 & 0 & 0 \\
0 & 0 & 0 & 0 & 0 & 0 & 0 & 0 & 0 & 0 & 0 & 0 & 0 & 1 & 0 & 0 & 2 & 0 & 1 \\
0 & 0 & 0 & 0 & 0 & 0 & 0 & 0 & 0 & 0 & 0 & 1 & 0 & 0 & 0 & 0 & 0 & 0 & 1 \\
0 & 0 & 0 & 0 & 0 & 0 & 0 & 0 & 0 & 0 & 0 & 0 & 0 & 0 & 1 & 2 & 0 & 1 & 0 \\
0 & 0 & 0 & 0 & 0 & 0 & 0 & 0 & 0 & 0 & 0 & 0 & 1 & 0 & 0 & 0 & 0 & 1 & 0 \\
0 & 0 & 0 & 1 & 0 & 0 & 0 & 0 & 0 & 0 & 0 & 0 & 0 & 1 & 1 & 0 & 0 & 0 & 0 \\
0 & 0 & 0 & 0 & 1 & 0 & 0 & 0 & 0 & 0 & 0 & 0 & 0 & 0 & 0 & 0 & 0 & 0 & 0 \\
0 & 0 & 0 & 0 & 0 & 1 & 0 & 0 & 0 & 0 & 0 & 0 & 0 & 0 & 0 & 0 & 0 & 0 & 0 \\
0 & 0 & 0 & 0 & 0 & 0 & 1 & 0 & 0 & 0 & 0 & 0 & 0 & 0 & 0 & 0 & 0 & 0 & 0
\end{pmatrix}$$

and taking mass action kinetics, the vector of mass action monomials reads:

$$\psi(c) = \left(c_1, c_2, c_3, c_{10}, c_{11}, c_{12}, c_{13}, c_4, c_5, c_2c_4, c_1c_5, c_3c_7, c_3c_9, c_6c_{10}, c_8c_{10}, c_8^2, c_6^2, c_8c_9, c_6c_7\right)^T.$$

The stoichiometric matrix is:

$$S = \begin{pmatrix} 1 & -1 & 0 & 0 & 0 & 0 & 1 & -1 & 0 & 0 & 0 & 0 & 0 & 0 & 0 & 0 & 0 \\ 0 & 0 & -1 & 1 & -1 & 1 & 0 & 0 & 0 & 0 & 0 & 0 & 0 & 0 & 0 & 0 & 0 \\ 0 & 0 & 0 & 0 & 1 & -1 & -1 & 1 & -1 & 1 & 1 & 0 & 0 & 0 & 0 & 0 & 0 \\ -1 & 1 & 0 & 0 & -1 & 1 & 0 & 0 & 0 & 0 & 0 & 0 & 0 & 0 & 0 & 0 & 0 \\ 0 & 0 & 1 & -1 & 0 & 0 & 1 & -1 & 0 & 0 & 0 & 0 & 0 & 0 & 0 & 0 & 0 \\ 0 & 0 & 0 & 0 & 0 & 0 & 0 & 0 & 0 & 0 & 0 & -1 & 0 & 0 & 2 & 0 & 1 \\ 0 & 0 & 0 & 0 & 0 & 0 & 0 & 0 & -1 & 1 & 0 & 0 & 0 & 0 & 0 & 0 & 1 \\ 0 & 0 & 0 & 0 & 0 & 0 & 0 & 0 & 0 & 0 & 0 & 0 & 1 & -2 & 0 & -1 & 0 \\ 0 & 0 & 0 & 0 & 0 & 0 & 0 & 0 & 0 & 0 & 1 & 0 & 0 & 0 & 0 & -1 & 0 \\ 0 & 0 & 0 & 0 & 0 & 0 & 0 & 0 & 1 & -1 & -1 & -1 & 1 & 0 & 0 & 0 & 0 \\ 0 & 0 & 0 & 0 & 0 & 0 & 0 & 0 & 0 & 0 & 0 & 1 & -1 & 0 & 0 & 0 & 0 \\ 0 & 0 & 0 & 0 & 0 & 0 & 0 & 0 & 0 & 0 & 0 & 0 & 0 & 1 & -1 & 0 & 0 \\ 0 & 0 & 0 & 0 & 0 & 0 & 0 & 0 & 0 & 0 & 0 & 0 & 0 & 0 & 0 & 1 & -1 \end{pmatrix}.$$

The rank of the matrix S (and therefore the dimension of the stoichiometric subspace) is $s = 9$. The deficiency of the network is:

$$\delta = 19 - 7 - 9 = 3,$$

and the dimension of the equilibrium manifold:

$$\lambda = 13 - 9 = 4.$$

The network is under-dimensioned ($\lambda > \delta$). There are four mass conservation laws, defined by $W(c, \sigma) = 0$, where $W(c, \sigma) = B^T c - \sigma$, $\sigma = B^T c$, and

$$B^T = \begin{pmatrix} 1 & 0 & 1 & 1 & 0 & 0 & 0 & 0 & 0 & 1 & 1 & 0 & 0 \\ 0 & 1 & 1 & 0 & 1 & 0 & 0 & 0 & 0 & 1 & 1 & 0 & 0 \\ 0 & 0 & 0 & 0 & 0 & 1 & 0 & 1 & 0 & 0 & 1 & 2 & 1 \\ 0 & 0 & 0 & 0 & 0 & 0 & 1 & 0 & 1 & 1 & 1 & 0 & 1 \end{pmatrix}.$$

The matrix Λ associated to the linkage classes reads:

$$\Lambda^T = \begin{pmatrix} 1 & 0 & 0 & 0 & 0 & 0 & 0 & 1 & 0 & 0 & 0 & 0 & 0 & 0 & 0 & 0 & 0 & 0 & 0 \\ 0 & 1 & 0 & 0 & 0 & 0 & 0 & 0 & 1 & 0 & 0 & 0 & 0 & 0 & 0 & 0 & 0 & 0 & 0 \\ 0 & 0 & 1 & 0 & 0 & 0 & 0 & 0 & 0 & 1 & 1 & 0 & 0 & 0 & 0 & 0 & 0 & 0 & 0 \\ 0 & 0 & 0 & 1 & 0 & 0 & 0 & 0 & 0 & 0 & 0 & 1 & 1 & 0 & 0 & 0 & 0 & 0 & 0 \\ 0 & 0 & 0 & 0 & 1 & 0 & 0 & 0 & 0 & 0 & 0 & 0 & 0 & 1 & 1 & 0 & 0 & 0 & 0 \\ 0 & 0 & 0 & 0 & 0 & 1 & 0 & 0 & 0 & 0 & 0 & 0 & 0 & 0 & 0 & 1 & 1 & 0 & 0 \\ 0 & 0 & 0 & 0 & 0 & 0 & 1 & 0 & 0 & 0 & 0 & 0 & 0 & 0 & 0 & 0 & 0 & 1 & 1 \end{pmatrix}.$$

Starting from the stoichiometric matrix Y and the matrix Λ we compute a basis for the deficiency subspace as:

$$\omega = \begin{pmatrix} -1 & 1 & 0 & 0 & 0 & 0 & 0 & 1 & -1 & -1 & 1 & 0 & 0 & 0 & 0 & 0 & 0 & 0 & 0 \\ 0 & 0 & 0 & 0 & 0 & 0 & 0 & 0 & 0 & 0 & 0 & 0 & 0 & -2 & 2 & -1 & 1 & 0 & 0 \\ 0 & 0 & 0 & 0 & 0 & 0 & 0 & 0 & 0 & 0 & 0 & -1 & 1 & -1 & 1 & 0 & 0 & -1 & 1 \end{pmatrix}^T,$$

and therefore we can express $A\psi$ as:

$$\begin{aligned}
k_{1\,8}\psi_1 - k_{8\,1}\psi_8 &= \alpha_1 \\
k_{2\,9}\psi_2 - k_{9\,2}\psi_9 &= -\alpha_1 \\
k_{3\,10}\psi_3 - k_{10\,3}\psi_{10} &= -\alpha_1 \\
k_{3\,11}\psi_3 - k_{11\,3}\psi_{11} &= \alpha_1 \\
k_{4\,12}\psi_4 - k_{12\,4}\psi_{12} &= -\alpha_3 \\
k_{4\,13}\psi_4 &= \alpha_3 \\
-k_{14\,5}\psi_{14} &= -2\alpha_2 - \alpha_3 \\
k_{5\,15}\psi_5 &= 2\alpha_2 + \alpha_3 \\
-k_{16\,6}\psi_{16} &= -\alpha_2 \\
k_{6\,17}\psi_6 &= \alpha_2 \\
-k_{18\,7}\psi_{18} &= -\alpha_3 \\
k_{7\,19}\psi_7 &= \alpha_3.
\end{aligned}$$

Substituting the elements of the vector ψ by the concentration monomials we obtain the equations defining the locus of equilibria:

$$\begin{aligned}
k_{1\,8}c_1 - k_{8\,1}c_4 - \alpha_1 &= 0 \\
k_{2\,9}c_2 - k_{9\,2}c_5 + \alpha_1 &= 0 \\
k_{3\,10}c_3 - k_{10\,3}c_2c_4 + \alpha_1 &= 0 \\
k_{3\,11}c_3 - k_{11\,3}c_1c_5 - \alpha_1 &= 0 \\
k_{4\,12}c_{10} - k_{12\,4}c_3c_7 + \alpha_3 &= 0 \\
k_{4\,13}c_{10} - \alpha_3 &= 0 \\
-k_{14\,5}c_6c_{10} + 2\alpha_2 + \alpha_3 &= 0 \\
k_{5\,15}c_{11} - 2\alpha_2 - \alpha_3 &= 0 \\
-k_{16\,6}c_8^2 + \alpha_2 &= 0 \\
k_{6\,17}c_{12} - \alpha_2 &= 0 \\
-k_{18\,7}c_8c_9 + \alpha_3 &= 0 \\
k_{7\,19}c_{13} - \alpha_3 &= 0.
\end{aligned}$$

We can express the concentrations as:

$$
\begin{aligned}
c_{12} &= \alpha_2/k_{6\,17} \\
c_8 &= (\alpha_2/k_{16\,6})^{1/2} \\
c_{13} &= \alpha_3/k_{7\,19} \\
c_9 &= \alpha_3 c_8/k_{18\,7} \\
c_{11} &= 2\alpha_2/k_{5\,15} + \alpha_3/k_{5\,15} \\
c_{10} &= \alpha_3/k_{4\,13} \\
c_6 &= (2\alpha_2 + \alpha_3)/(c_{10}k_{14\,5}) \\
c_4 &= (k_{1\,8}c_1 - \alpha_1)/k_{8\,1} \\
c_2 &= (-k_{3\,10}k_{11\,3}c_1/(k_{9\,2}k_{3\,11}) - 1 - k_{3\,10}/k_{3\,11})\alpha_1/(k_{3\,10}k_{11\,3}k_{2\,9}c_1/(k_{9\,2}k_{3\,11}) - k_{10\,3}(k_{1\,8}c_1 - \alpha_1)/k_{8\,1}) \text{[illegible]} \\
c_5 &= (\alpha_1 + k_{2\,9}c_2)/k_{9\,2} \\
c_3 &= (k_{11\,3}c_1(\alpha_1 + k_{2\,9}c_2)/k_{9\,2}) + \alpha_1)/k_{3\,11} \\
c_7 &= (\alpha_3 + k_{4\,12}c_{10})/(k_{12\,4}c_3)
\end{aligned}
$$

The matrix G in Eq. (13) in the main text reads (next page):

$$
G = \begin{pmatrix}
k_{1\,8} & 0 & 0 & -k_{8\,1} & 0 & 0 & 0 & 0 & 0 & 0 & 0 & 0 & 0 & -1 & 0 & 0 \\
0 & k_{2\,9} & 0 & 0 & -k_{9\,2} & 0 & 0 & 0 & 0 & 0 & 0 & 0 & 0 & 1 & 0 & 0 \\
0 & -k_{10\,3}c_4 & k_{3\,10} & -k_{10\,3}c_2 & 0 & 0 & 0 & 0 & 0 & 0 & 0 & 0 & 0 & 1 & 0 & 0 \\
-k_{11\,3}c_5 & 0 & k_{3\,11} & 0 & -k_{11\,3}c_1 & 0 & 0 & 0 & 0 & 0 & 0 & 0 & 0 & -1 & 0 & 0 \\
0 & 0 & -k_{12\,4}c_7 & 0 & 0 & 0 & -k_{12\,4}c_3 & 0 & 0 & k_{4\,12} & 0 & 0 & 0 & 0 & 0 & 1 \\
0 & 0 & 0 & 0 & 0 & 0 & 0 & 0 & 0 & k_{4\,13} & 0 & 0 & 0 & 0 & 0 & -1 \\
0 & 0 & 0 & 0 & 0 & -k_{14\,5}c_10 & 0 & 0 & 0 & -k_{14\,5}c_6 & 0 & 0 & 0 & 0 & 2 & 1 \\
0 & 0 & 0 & 0 & 0 & 0 & 0 & 0 & 0 & 0 & k_{5\,15} & 0 & 0 & 0 & -2 & -1 \\
0 & 0 & 0 & 0 & 0 & 0 & 0 & -2k_{16\,6}c_8 & 0 & 0 & 0 & 0 & 0 & 0 & 1 & 0 \\
0 & 0 & 0 & 0 & 0 & 0 & 0 & 0 & 0 & 0 & 0 & k_{6\,17} & 0 & 0 & -1 & 0 \\
0 & 0 & 0 & 0 & 0 & 0 & 0 & -k_{18\,7}c_9 & -k_{18\,7}c_8 & 0 & 0 & 0 & 0 & 0 & 0 & 1 \\
0 & 0 & 0 & 0 & 0 & 0 & 0 & 0 & 0 & 0 & 0 & 0 & k_{7\,19} & 0 & 0 & -1 \\
0 & 1 & 1 & 0 & 1 & 0 & 0 & 0 & 0 & 1 & 1 & 0 & 0 & 0 & 0 & 0 \\
1 & 0 & 1 & 1 & 0 & 0 & 0 & 0 & 0 & 1 & 1 & 0 & 0 & 0 & 0 & 0 \\
0 & 0 & 0 & 0 & 0 & 1 & 0 & 1 & 0 & 0 & 1 & 2 & 1 & 0 & 0 & 0 \\
0 & 0 & 0 & 0 & 0 & 0 & 1 & 0 & 1 & 1 & 1 & 0 & 1 & 0 & 0 & 0]
\end{pmatrix}
$$

Table 7.1: Optimum set of parameters for the (closed) network in Fig. 6A.

$k_{8\,1}$	$k_{2\,9}$	$k_{10\,3}$	$k_{11\,3}$	$k_{12\,4}$	$k_{4\,12}$	$k_{4\,13}$	$k_{14\,5}$	$k_{5\,15}$	$k_{16\,6}$	$k_{6\,17}$	$k_{18\,7}$	$k_{7\,19}$	α_1	α_2	α_3	c_1
72.0528	14.5045	47.5051	23.4905	26.0821	30.2955	92.0138	90.5432	39.3903	72.6030	94.3907	95.0770	29.0424	-0.3091	976.6046	829.3275	0.9685

We formulate the optimization problem defined by Eq. (15) in the main text, to find parameter vectors for which the determinant of matrix G becomes zero. We minimize the objective function $F_{def} = det(G)^2$ using as decision vector:

$$\begin{aligned} \mathrm{x} =(& k_{81},\ k_{29},\ k_{10\,3},\ k_{11\,3},\ k_{12\,4},\ k_{4\,12},\ k_{4\,13},\ k_{14\,5},\ k_{5\,15},\ k_{16\,6},\ k_{6\,17}, \ldots \\ & k_{18\,7},\ k_{7\,19},\ \alpha_1,\ \alpha_2,\ \alpha_3,\ c_1). \end{aligned}$$

In order to incorporate current knowledge about kinetic relations in the pathway we considered fixed $k_{1\,8} = 1$, $k_{9\,2} = 0.01$, $k_{3\,10} = 0.4$ and $k_{3\,11} = 0.1$. We found a decision vector for which the objective function vanishes (see Table 7.1). At this point, we start a continuation of equilibrium and confirm that the system undergoes a saddle-node bifurcation. In fact, the network has capacity for bistability (see Figs 7.1 and 7.2).

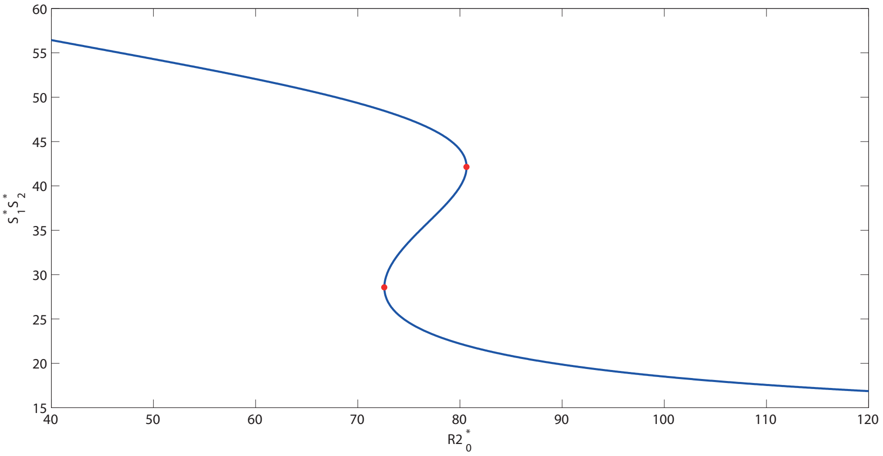

Figure 7.1: Bifurcation diagram for the receptor complex formation and STAT signaling network. A continuation of equilibria is started from the optimal point found by our algorithm (here we vary the total concentration of receptor subunit $R2_0$ in forward and backward directions). Tangent bifurcation points are indicated in red.

The Matlab code is provided in S1 file (`Matlab_code/IFN_merged`).

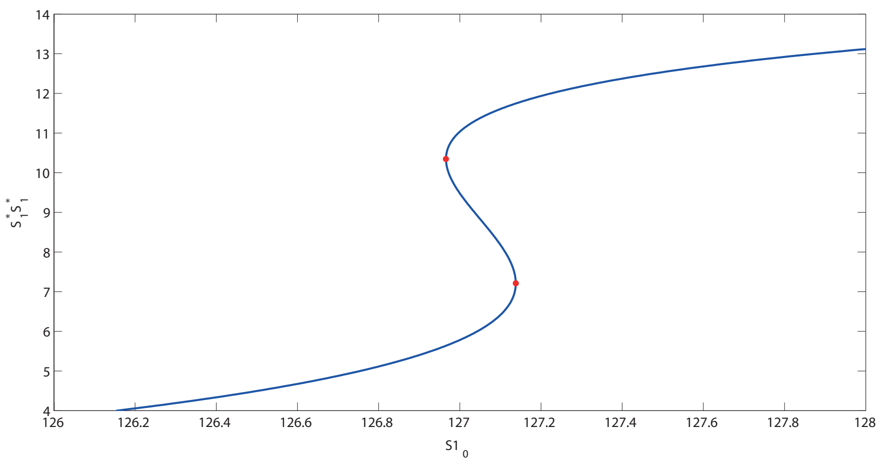

Figure 7.2: Bifurcation diagram for the receptor complex formation and STAT signaling network. A continuation of equilibria is started from the optimal point found by our algorithm (here we vary the total concentration of STAT1 $S1_0$ in forward and backward directions). Tangent bifurcation points are indicated in red.

Chapter 8

STAT activation dynamics upon IFN stimulation

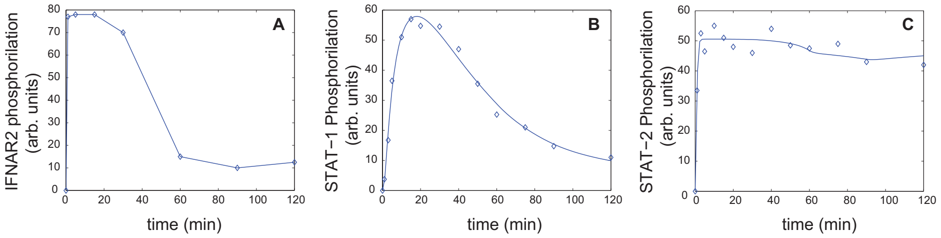

Figure 8.1: STATs dynamics after IFN stimulation in WISH cells treated with 500 pM (saturated doses) of IFNα_2. (A) Measured time series of phosphorylated IFNAR2, (B) fit of the model to the measured time series of phosphorylated STAT1 and (C) fit of the model to the measured time series of phosphorylated STAT2.

The IFN-STAT signaling network model which we use for the analysis is compatible with the STAT activation dynamics observed upon IFN stimulation, as it can be deduced from the fit of the model to experimental data (Fig. 8.1). Experimental data were provided by Ignacio Moraga and Sandra Pellegrini from the IFNaction Consortium.

Bibliography

[1] Kuznetov, Yu. A. (2009). Five lectures on numerical bifurcation analysis by Kuznetsov, Utrecht University, NL.

[2] Kuznetov, Yu. A. (1998). Elements of applied bifurcation theory, Second Edition, Springer, NY.

[3] Seydel, R (2006). Practical bifurcation and stability analysis, Springer.

[4] Chicone, C (2006). Ordinary differential equations with applications, Springer, NY.

[5] Dhooge, A, Govaerts, W and Kuznetsov, Yu A (2003). MatCont: A MATLAB package for numerical bifurcation analysis of ODEs. ACM transactions on mathematical software, 29:141–164.

[6] Otero-Muras I, Banga JR, Alonso AA (2012). Characterizing multistationarity regimes in biochemical reaction networks. PLoS ONE 7(7): e39194.

[7] Egea JA, Rodriguez-Fernandez M, Banga JR and Marti R (2007). Scatter search for chemical and bioprocess optimization. *J. Global Optim.* 37(3):481–503.

[8] Egea JA, Henriques D, Cokelaer T, Villaverde AF, MacNamara A, Danciu DP, Banga JR and Saez-Rodriguez, J 2014. MEIGO: an open-source software suite based on metaheuristics for global optimization in systems biology and bioinformatics BMC Bioinformatics, 15:136.
